# A multi-functional hypoxia/esterase dual stimulus responsive and hyaluronic acid-based nanomicelle for targeting delivery of chloroethylnitrosouea

**DOI:** 10.1186/s12951-023-02062-3

**Published:** 2023-08-23

**Authors:** Duo Li, Ting Ren, Yunxuan Ge, Xiaoli Wang, Guohui Sun, Na Zhang, Lijiao Zhao, Rugang Zhong

**Affiliations:** Beijing Key Laboratory of Environmental & Viral Oncology, Faculty of Environment & Life, Beijing University of Technology, Beijing, 100124 China

**Keywords:** Carmustine, *O*^6^-alkylguanine-DNA alkyltransferase, Drug delivery, Stimulus-responsive nanocarrier

## Abstract

Carmustine (BCNU), a vital type of chloroethylnitrosourea (CENU), inhibits tumor cells growth by inducing DNA damage at *O*^6^ position of guanine and eventually forming dG-dC interstrand cross-links (ICLs). However, the clinical application of BCNU is hindered to some extent by the absence of tumor selectivity, poor stability and *O*^6^-alkylguanine-DNA alkyltransferase (AGT) mediated drug resistance. In recent years, tumor microenvironment has been widely utilized for advanced drug delivery. In the light of the features of tumor microenvironment, we constructed a multifunctional hypoxia/esterase-degradable nanomicelle with AGT inhibitory activity named HACB NPs for tumor-targeting BCNU delivery and tumor sensitization. HACB NPs was self-assembled from hyaluronic acid azobenzene AGT inhibitor conjugates, in which *O*^6^-BG analog acted as an AGT inhibitor, azobenzene acted as a hypoxia-responsive linker and carboxylate ester bond acted as both an esterase-sensitive switch and a connector with hyaluronic acid (HA). The obtained HACB NPs possessed good stability, favorable biosafety and hypoxia/esterase-responsive drug-releasing ability. BCNU-loaded HACB/BCNU NPs exhibited superior cytotoxicity and apoptosis-inducing ability toward the human uterine cervix carcinoma HeLa cells compared with traditional combined medication of BCNU plus *O*^6^-BG. In vivo studies further demonstrated that after a selective accumulation in the tumor site, the micelles could respond to hypoxic tumor tissue for rapid drug release to an effective therapeutic dosage. Thus, this multifunctional stimulus-responsive nanocarrier could be a new promising strategy to enhance the anticancer efficacy and reduce the side effects of BCNU and other CENUs.

**Supplementary Information:**

The online version contains supplementary material available at 10.1186/s12951-023-02062-3.

## Introduction

As a classical type of chloroethylnitrosoureas (CENUs), carmustine (BCNU) has been demonstrated to be efficient against various tumors including brain tumors, bronchogenic carcinoma and carcinoma of the female genitalia [1–3]. It exerts antitumor activity through alkylating guanine bases at the *O*^6^ position followed by covalently connecting with the complementary cytosine to form interstrand cross-links (ICLs) [4, 5]. The DNA ICLs hinders double-strand separation during DNA replication or transcription and consequently inhibits the growth of tumor cells eventually killing tumor cells [6]. Unfortunately, the shortcomings of BCNU, such as short elimination half-life, deficient selectivity and poor water-solubility, increase the frequency of systemic administration of BCNU and result in serious side effects, including hepatic toxicity, myelosuppression, and pulmonary fibrosis [7]. Besides, the occurrence of drug resistance is one of the main factors leading to the failure of BCNU chemotherapy. *O*^6^-Alkylguanine-DNA alkyltransferase (AGT), a DNA-repair enzyme, can restore the BCNU-induced DNA damage by transferring the alkyl groups from the *O*^6^-position of guanine to the cysteine 145 (Cys145) residue in the active site [8, 9]. This repair of the *O*^6^-alkylated guanine blocks the formation of ICLs, resulting in resistance of cells to BCNU, especially in the tumor tissues with high expression levels of AGT [10].

*O*^6^-Benzylguanine (*O*^6^-BG) is an effective AGT inactivator, which is often used as chemotherapeutic adjuvants to improve the therapeutic efficacy of BCNU. It acts as a pseudosubstrate to transfers its benzyl group to the Cys145 residue at the active site of AGT, resulting in the ubiquitination and degradation of AGT enzyme. A large amount of research has revealed that *O*^6^-BG administration could effectively reverse the resistance of cancer cells to BCNU both in vitro and in vivo [11–14] In recent years, several combi-nitrosourea prodrugs have been synthesized to improve the treatment efficacy of CENUs, which combine the CENU pharmacophore and the *O*^6^-BG analog moiety in one molecule [15–18]. Nevertheless, both the traditional combination therapy and the combi-nitrosourea prodrugs have a fatal disadvantage of inability to target tumors tissue. They were observed to act on tumor cells and normal cells indiscriminately, consequently resulting in serious side effects including bone marrow toxicity and even secondary tumors [19]. Thus, new approaches are required to improve the therapeutic efficiency and reduce the side effects by targeted delivery of CENUs to tumor regions.

Tumor hypoxia is a unique feature of most solid tumors stemming from the rapid proliferation of tumor cells, abnormal vasculatures, and high interstitial pressures of solid tumors [20, 21]. Due to its important role in tumor metabolism, hypoxia has been considered a critical characteristic for developing novel tumor therapeutics [22]. Last decade, hypoxia-activated prodrugs, which could be transformed from nontoxic prodrug molecules into antitumor agents by reduction under hypoxia, have become a promising strategy in precision treatment of tumors [23, 24]. In our previous study, an azobenzene-based combi-nitrosourea prodrug, named AzoBGNU, was synthesized to selectively sensitize the oxygen-deficient tumor cells to the chloroethylating agent by achieving hypoxia-targeting inhibition of AGT. As expected, AzoBGNU showed higher proliferation inhibition against DU145 cells with high levels of AGT expression than free nimustine under hypoxic conditions [10]. However, the in vivo application of the combi-molecular prodrugs might be limited by its poor solubility as a result of the complicated chemical modification [25]. Moreover, the high synthetic burden for obtaining the final prodrug was also a restriction of the development of combi-nitrosourea prodrugs from the viewpoint of pharmacoeconomics [22]. Thus, it is necessary to discover a novel approach for overcoming the limitations of these hypoxia-responsive nitrosourea prodrugs.

Nanomicelles constructed by the self-assembly behavior of polymer-drug conjugates possess advantages of simple process, high drug-loading and controllable release, displaying great potential in tumor-targeted delivery [26]. Hyaluronic acid (HA), a natural polysaccharide, was widely utilized in biomedical applications due to its biocompatibility and strong hydrophilicity. Additionally, owing to the specifical combination with the CD44 receptors overexpressed on the surface of cancer cells, HA can be used for the construction of targeting nano delivery system of chemotherapies [27, 28]. Li et al. [29] reported that HA modification resulted in a significant increase of the amount of cisplatin-loaded nano-assembly internalized by 4T1 cells. Sun et al. [30] described that HA modification could enhance the tumor-specific accumulation of nanoparticles via CD44-mediated pathway. Therefore, we hypothesized that HA could be covalently bonded with the azobenzene-modified *O*^6^-BG conjugates for the construction of a hypoxia-responsive delivery system for targeted delivery of BCNU. However, a critical issue faced by hypoxia-responsive delivery systems is that because of the heterogeneity of hypoxia inside tumors, the loaded chemotherapy cannot be rapidly released in oxygen-rich tumor cells nearby blood vasculatures, which reduces the sensitivity of the nanocarrier to tumor environment and thereby decreases drug efficacy [26, 31]. As a result, hypoxia-responsive nanocarriers are needed to be optimized to achieve desirable drug concentrations in whole tumor tissue. Based on the high-level expression of esterase in tumor cells, an esterase-sensitive chemical bond, carboxylate ester bond, was introduced into the structure of the nanocarriers to enhance the efficacy of targeted drug release [32, 33]. The stability of the nanocarriers can be destroyed because of the hydrolyzation of the ester bond by the intracellular esterase [34, 35]. Su et al. [36] developed a prodrug conjugate (ICG-HA-PTX) by covalently linking indocyanine green derivative and PTX to HA through an ester bond, which was disrupted by the overexpressed esterase, leading to rapid release of PTX and ICG.

**Scheme 1:**
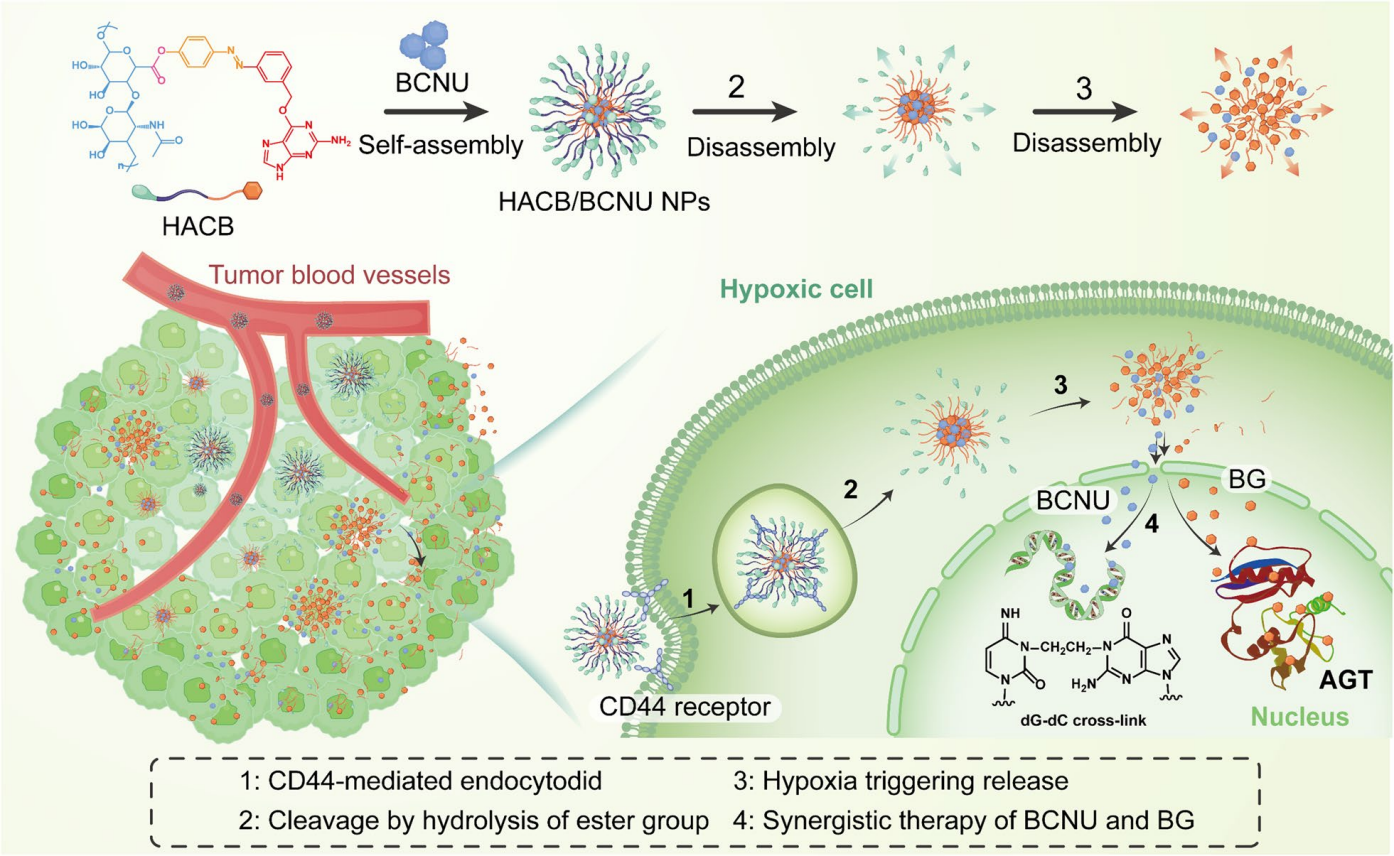
Schematic illustration for the synthesis and antitumor mechanism of HACB/BCNU NPs

In this study, we successfully constructed an amphiphilic conjugate by covalently linking HA and the azobenzene-modified *O*^6^-BG derivative (BG-AZO-COOH) through an ester bond, forming HA-AZO-COO-BG (abbreviated as HACB). In an aqueous solution, BCNU was encapsulated within the synthesized HACB to form micelles (HACB/BCNU NPs) via self-assembly behavior. In the micelles, BCNU itself was not modified, but rather hosted within the hydrophobic core of the stimuli-responsive drug-delivery system. As displayed in Scheme 1, after reaching the tumor site, the ester bonds are cleaved by the overexpressed esterase leading to rapid release of BCNU. Under the oxygen-deficient environment of tumor, *O*^6^-(3-amino-benzyl) guanine acting as an AGT inhibitor is yielded via the reduction of the azo bond to aniline, and BCNU is fully released simultaneously, thus achieving an enhanced anticancer therapeutic efficacy. In vitro and in vivo investigations were carried out to confirm the dual responsive release behavior and the anticancer activity of HACB/BCNU NPs. This study is expected to contribute to the development of novel tumor-targeting nitrosourea chemotherapies with enhanced efficacy and reduced side effect.

## Materials and methods

### Materials

3-Aminobenzylalcohol, phenol and sodium hydride were purchased from Energy Chemical (Shanghai, China). 1-Methylpyrrolidine and 6-chloropurine were obtained from Bidepharm (Shanghai, China). Hyaluronic acid (HA, MW 5000 Da) was brought from HEOWNS Co., Ltd. (Tianjin, China). *N*-Hydroxysuccinimide (NHS) and 1-ethyl-3-(3-(dimethylamino) propyl) carbodiimide (EDC) were acquired from Aladdin Reagent Co., Ltd. (Shanghai, China). Reduced nicotinamide adenine dinucleotide phosphate oxidase (NADPH) and coumarin-6 (Cou6) were got from Sigma-Aldrich (St. Louis, United States). BCNU was brought from Tokyo Chemical Industry Co., Ltd. (Tokyo, Japan). Esterase from porcine liver was provided by MACKLIN Co., Ltd. (Shanghai, China). Fetal bovine serum (FBS) was bought from Zhejiang Biological technology stock Co., Ltd. (Zhejiang, China). Rat liver microsomes were obtained from PrimeTox (Wuhan, China). Roswell Park Memorial Institute 1640 Medium (1640) and penicillin–streptomycin solution were purchased from HyClone (Logan City, USA). 3-(4,5-dimethyl-2-thiazolyl)-2,5-diphenyl-2-H-tetrazolium bromide (MTT) was provided by Beyotime Biotechnology Co., Ltd (Shanghai, China). Hoechst33342/PI apoptosis detection kit, Annexin V-FITC/PI apoptosis detection kit and Alamar blue were acquired from Solarbio Life Science (Beijing, China). All other reagents were purchased from Beijing Chemicals Co. (Beijing, China) and J&K Scientific Ltd. (Shanghai, China).

### Cell culture

The human uterine cervix carcinoma cells (HeLa), the human non-small cell lung cancer cells (A549), the hepatocellular carcinoma cells (SMMC-7721) and mouse brain endothelial cells (b End. 3) were purchased from Peking Union Medical College (Beijing, China). All cells were cultured in RPMI-1640 medium containing 10% FBS, 100 U/mL of penicillin and 100 µg/mL of streptomycin at 37 ℃ using a humidified 5% CO_2_ incubator (Thermo Fisher Scientific Inc., Waltham, MA, USA). Hypoxic conditions in cell culture were generated by a hypoxia anaerobic incubator chamber (Thermo 1025, Thermo Fisher Scientific, Waltham, MA, USA) supplemented with an appropriate humidified gas mixture containing 1% O_2_ and 5% CO_2_ balanced with N_2_.

### Animals

BALB/c nude mice (female, 4–6 weeks) were purchased from Vital River Laboratory Animal Technology Co. Ltd (Beijing, China). All animal experiments were conducted under a protocol approved by Beijing University of Technology Institutional Animal Care and Use Committee. Subcutaneous tumor models were planted by injecting 1 × 10^7^ HeLa cells (100 *µ*L) into the left side of each mouse near the armpit. Tumor volume (V) was determined by measuring the length (L) and the width (W), and was calculated according to Eq. (1).

1 $$\mathrm{V}=\frac{1}{2}L\times {W}^{2}$$

### Synthesis of HACB Conjugates

#### Synthesis of 4-((3-(hydroxymethyl)phenyl) diazenyl) phenol (*a*)

3-Aminobenzylalcohol (123 mg) was dissolved in 800 *µ*L deionized water. Then, 800 *µ*L hydrochloric acid and 1 mL cold sodium nitrite solution (2 mmol/L) were dropwise added to the above solution stirring for 20 min at 0–4 ℃. After that, 96 *µ*L phenol was added to the solution stirring for 24 h at 0–4 ℃. Then the solution was extracted with distilled water/ethyl acetate (1:1). The organic layers were collected, dehydrated with sodium sulfate and evaporated to get a bronzing solid (0.19 mmol, 19% yield). ^1^H NMR (400 MHz, DMSO-*d*_6_) δ: 10.26 (s, 1H), 7.69–7.83 (m, 4H), 7.43–7.53 (m, 2H), 6.94–6.96 (d, 2H), 5.35–5.38 (t, 1H), 4.60–4.61 (d, 2H). ^13^C NMR (101 MHz, DMSO-*d*_6_) δ: 161.38, 152.56, 145.72, 144.47, 129.48, 128.85, 125.25, 121.69, 119.68, 116.41, 63.00. IR (KBr) υ: 3479.41, 3184.31 (OH), 2881.61 (CH_2_), 1593 (C=C), 1506.40 (N=N), 1145.05 (C–N), 1145.05 (C–O), 838.95 (=C–H), 694.11 (C–H).

#### Synthesis of 1-(2-amino-9H-purin-6-yl)-1-methylpyrrolidine-1-chloride (**b**)

Compound **b** was synthesized according to the method described by Keppler et al. with modification [37] Briefly, 2-amino-6-chloropurine (2 g) was dissolved in *N*, *N*-dimethylformamide (DMF). Then, 1-methylpyrrolidine (3 mL) was added into the DMF solution to react under stirring for 20 h at room temperature. Subsequently, acetone (3 mL) was added to the above solution for precipitation. Then, the precipitates were filtered and washed twice by ether to get a white solid (7.08 mmol, 60% yield). ^1^H NMR (400 MHz, DMSO-*d*_6_) δ: 13.45 (s, 1H), 8.35 (s, 1H), 7.12 (s, 2H), 4.63–4.57 (m, 2H), 4.01–3.95 (m, 2H), 3.66 (s, 3H), 2.34–2.20 (m, 2H), 2.11–2.00 (m, 2H). ^13^C NMR (101 MHz, DMSO-*d*_6_) δ: 161.30, 159.52, 157.63, 150.63, 141.81, 117.07, 65.23, 51.50, 22.37. IR (KBr) υ: 3290.57 (NH_2_), 3350.92 (NH), 3184.60 (=C–H), 2897.59 (CH_2_), 1631.80 (C=N), 1564.10 (C=C), 1366.87 (C–C), 1293.28 (C–N), 816.40 (=C–H), 789.91 (N–H), 625.06 (C–H).

#### Synthesis of 4-((3-(((2-amino-9H-purin-6-yl) oxy) methyl) phenyl) diazenyl) phenol (*c*)

Compound **a** (580 mg) was dissolved in 10 mL DMF, to which 600 mg potassium *tert*-butoxide and 300 mg compound **b** were added. Then, the mixture was stirred under a nitrogen atmosphere for 6 h at room temperature. Subsequently, 1 mL of deionized water containing 100 *µ*L glacial acetic acid was added to the above solution. Then, the mixture was extracted with ethyl acetate/deionized water (1:1). The organic layers were dried with anhydrous sodium sulfate and concentrated under a vacuum. Finally, the crude product was purified by column chromatography with dichloromethane/methanol (50:1–10:1) as an eluent to obtain a yellow solid (1.28 mmol, 51% yield). ^1^H NMR (400 MHz, DMSO-*d*_6_) δ: 12.48 (s, 1H), 10.37 (s, 1H), 7.93 (s, 1H), 7.84–7.79 (m, 4H), 7.64–7.57(m, 2H), 6.96–6.94 (d, 2H), 6.36 (s, 2H), 5.60 (s, 2H). ^13^C NMR (101 MHz, DMSO-*d*_6_) δ: 161.57, 160.13, 152.67, 145.67, 138.73, 138.41, 130.64, 129.90, 125.39, 122.26, 122.05, 116.43, 66.73. IR (KBr) υ: 3683.45 (OH), 3487.84 (NH_2_), 3340.02 (NH), 2802.47 (CH), 1587.03 (C=C), 1501.92 (N=N), 1355.59 (C–C), 1283.92 (C–O–C), 1146.54 (C–N), 837.46 (=C–H), 786.69 (N–H), 628.41 (C–H).

#### Synthesis of HACB conjugates

An aliquot of HA (35 mg, MW =  ~ 5000 Da) was dissolved in distilled water (7 mg/mL) followed by addition of 15 mg EDC and 9 mg NHS, and the mixture was stirred at room temperature for 0.5 h to activate the carboxyl groups of HA. Then, 243 mg of compound **c** was added to the above solution and stirred at 40 ℃ for 48 h. The obtained crude product was then dialyzed by a dialysis bag (MWCO 3500 Da) against an excess amount of water/DMSO (1:3 to 1:1) for 1 day and against distilled water for another 2 days. The solution was filtered through a microporous membrane with 0.45 *µ*m pores followed by freezing and lyophilizing to obtain a yellowish fluffy powder, named HACB.

### Preparation of blank (HACB NPs) or BCNU-loaded micelles (HACB/BCNU NPs)

HACB/BCNU NPs were prepared by solvent volatilization method according to the literature with some modifications [38]. Briefly, a solution of BCNU in ethanol (100 mg/mL) was dropwise added into the aqueous solutions of HACB (25 mg/mL) at different BCNU/HACB ratios (0.5:10, 1:10, 2:10, 3:10, 4:10, w/w) with continuous stirring. For blank micelles, BCNU was omitted. Subsequently, the mixture was sonicated with a probe-type ultrasonicator (Autotune, SONICS Newtown, USA) in an ice bath for 15 min (400 W, working for 5 s, intermittent 2 s). After that, the solution was filtered through a microporous membrane with 0.45 *µ*m pores followed by lyophilizing and stored at − 20 ℃ until further use. For visualization, Cou6-loaded micelles (HACB/Cou6 NPs) were prepared using Cou6 instead of BCNU.

### Characterization of HACB NPs and HACB/BCNU NPs

Dynamic light scattering (DLS) measurements were performed to examine the hydrodynamic size, zeta-potential and poly-dispersity (PDI) of HACB NPs and HACB/BCNU NPs using a laser diffraction particle sizer (Nano-ZS, Malvern Instrument, UK). The optical absorption was determined with a UV–vis spectrophotometer (TU-1901, PERSEE, China). The morphology of the micelles was monitored using transmission electron microscopy (TEM, JEM-2100, JEOL, Japan). To investigate the stability of HACB NPs, the mean particle size of the micelles dissolved in H_2_O, PBS (pH 7.4) or DMEM with 10% FBS was measured at different time points (0, 6, 12, 24, 36 and 48 h).

The encapsulation efficiency (EE) and drug loading (DL) of HACB/BCNU NPs were determined by measuring the concentration of the loaded drugs using HPLC (U3000, Thermo Fisher Scientific, USA) with a C18 column (ZORBAX 5 µm C18, 4.6 × 250 mm, Agilent Technologies Inc., California, USA). Briefly, the total dispersoid was filtered with an Amicon Ultra 10 kD molecular weight centrifugal filter (Millipore, Billerica, MA, USA) to obtain filtrate, which contained the unencapsulated BCNU. Then, the total mixture of the drug was dissolved in ten-fold volume of methanol followed by filtering through 0.22 *µ*m membrane filters. The obtained filtrate was used for the determination of the total BCNU concentration in the solution. All the samples were analyzed by HPLC and the ultraviolet absorption was monitored at 230 nm for BCNU. The values of EE and DL of HACB/BCNU NPs were calculated according to Eqs. (2) and (3):

2 $$\mathrm{EE}=\frac{Weight \,of \,loaded \,BCNU}{Total \,weight \,of \,BCNU \,in \,the \,reaction \,solution }\times 100$$

3 $$\mathrm{DL}=\frac{Weight\, of \,loaded \,BCNU}{Total \,weight \,of \,micelles}\times 100$$

### Cleavage study of HACB NPs

#### Hypoxia-sensitivity of HACB NPs

To simulate the hypoxic reductive environment, 1 mM Na_2_S_2_O_4_ was added into the aqueous solution with HACB NPs (1 mg/mL) and the mixture was stirred continuously at 37 ℃. The change of the absorption peak of the azobenzene group in the reaction was recorded by a UV–vis spectrophotometer. Moreover, HACB NPs was incubated with or without Na_2_S_2_O_4_ for 1 h at 37 ℃, and then the hydrodynamic diameter distributions and the morphology were observed by DLS and TEM, respectively.

#### Esterase responsiveness of HACB NPs

HACB NPs dispersion at a concentration of 1 mg/mL was incubated with or without 30 U/mL porcine liver esterase at 37 ℃ under continuous shaking (100 rpm) for 1 h. After that, the hydrodynamic diameter distributions of the micelles were monitored by DLS, and the morphology was observed by TEM.

#### In vitro drug release profile

On account of the high instability of BCNU in solution, a Cou6 model was used to examine the in vitro release profile of the micelles. In brief, 2 mL HACB/Cou6 NPs dispersion (containing 2 mg Cou6) was placed into a dialysis bag (MWCO of 1 kDa), and then immersed in 40 mL release media containing 0.5% w/v sodium dodecyl sulfate, porcine liver esterase (0 or 30 U/mL), 790 *µ*g/mL rat liver microsomes and 100 µM NADPH. Air and Nitrogen gas were bubbled through the reaction mixture to create normoxic and hypoxic conditions, respectively. Then, each group was gently shaken at 37 ℃ at a speed of 100 rpm. At the predetermined time point, 0.5 mL of the mixture containing the released Cou6 was drawn for measurement and replaced with fresh medium to maintain a constant volume. Three independent repeats were performed for each sample (n = 3).

### Cellular uptake and intracellular release of HACB/Cou6 NPs

Hela cells were seeded in 12-well plates at a density of 1.5 × 10^5^/well and cultured for 24 h at 37 ℃. Then, cells were treated with Cou6 or HACB/Cou6 NPs (Cou6 concentration of 10 ng/mL) for 30 min and 2 h under normoxia and hypoxia. To demonstrate the assisted targeting effect of HA, the cells were pretreated with 5 mg/mL HA for 2 h before exposure to HACB/Cou6 NPs. After treatment, the cells were washed twice with PBS, stained by Hoechst 33342 (10 *µ*g/mL) for 15 min and observed by an IX-51 inverted fluorescence microscope (Olympus Corporation, Tokyo, Japan). To further reveal the intracellular distribution of HACB/Cou6 NPs, the intracellular fluorescence was observed under a confocal laser scanning microscope (CLSM, Nikon AX R, Tokyo, Japan).

### In vitro cytotoxicity assay

The cytotoxicity of HACB/BCNU NPs was investigated by an MTT assay. Generally, HeLa cells were seeded in 96-well plates at a density of 5 × 10^3^/well and cultured for 24 h at 37 ℃. Then, the cells were treated with BCNU and HACB/BCNU NPs (at equivalent BCNU concentrations of 10, 20, 50, 100, 200, 400, 600 and 1000 *µ*M) under normoxic and hypoxic conditions. For the combination-treated groups, the cells were pretreated with 40 *µ*M *O*^6^-BG for 2 h before exposure to BCNU. Moreover, HeLa cells and a normal cell line b End.3 cells were treated with HACB NPs (0.02, 0.04, 0.10, 0.21, 0.42, 0.83, 1.25 and 2.08 mg/mL) to evaluate the cytotoxicity of blank micelles. After a 24-h treatment, 10 *µ*L MTT solutions (5 mg/mL) were added in each well, and the cells were incubated in the dark for 4 h. Subsequently, the medium was removed and formazan crystals were dissolved in 150 *µ*L DMSO. Finally, the absorbance at 560 nm was determined by a Thermo Scientific Multiskan FC (Multiskan FC, Thermo Scientific, Waltham, MA, USA). The cell viability was calculated according to Eq. (4) as follows:

4 $$\mathrm{Cell \,viability }\left(\mathrm{\%}\right)={(OD}_{sample}-{OD}_{blank})/( {OD}_{control}-{OD}_{blank})$$

Here, the ${OD}_{sample}$ is the absorbance values of the drug-treated cells, ${OD}_{blank}$ is the absorbance values of blank only containing medium and ${OD}_{control}$ is the absorbance values of the cells without drug treatment.

### Live/dead cell staining assay

HeLa cells were seeded in 48-well plates at 8 × 10^4^ cells per well and incubated for 24 h. Afterward, the cells were exposed to BCNU and HACB/BCNU NPs based on BCNU concentrations of 0.1 mM or 0.3 mM under normoxic and hypoxic conditions for 24 h. The combination-treated groups were pretreated with 40 *µ*M *O*^6^-BG for 2 h before exposure to BCNU. After that, the cells were stained with 5 *µ*L Hoechst 33,342 and 5 *µ*L PI solution for 15 min. Finally, the cells were washed three times with PBS and observed with a fluorescent microscope (IX-51, Olympus Corporation, Tokyo, Japan).

### Colony formation assay

HeLa cells were seeded in 6-well plates at 800 cells per well and maintained for 24 h. Thereafter, the medium was replaced with fresh medium containing BCNU, BCNU + *O*^6^-BG or HACB/BCNU NPs (containing 0.05 mM BCNU) under normoxia or hypoxia. The dose of BCNU in each group was 0.05 mM. After treatment for 24 h, the medium was replaced with fresh medium followed by incubation for another 8 days. For the BCNU + *O*^6^-BG group, the cells were pretreated by 40 *µ*M *O*^6^-BG for 2 h before exposure to BCNU. The cell clones were stained with crystal violet for 10 min followed by washing three times with PBS for observation.

### Cell apoptosis

The apoptosis induced by HACB/BCNU NPs was evaluated by Annexin V-FITC/PI apoptosis detection kit. HeLa cells were seeded in 6-well plates at 4 × 10^5^ cells per well and incubated for 24 h. Later, the cells were exposed to BCNU or HACB/BCNU NPs (containing 0.1 mM or 0.3 mM BCNU) under normoxic or hypoxic conditions for 24 h. The combination-treated groups were pretreated by 40 *µ*M *O*^6^-BG for 2 h before BCNU exposure. Then, the cells were digested with trypsin without EDTA, centrifuged and washed twice with pre-cooled PBS. The cells were resuspended in a 1 × binding buffer and co-stained with Annexin V-FITC/PI kit for detection by FACS Calibur flow cytometer (BD Biosciences, San Jose, CA, USA).

### Cell wound-healing assay

Wound-healing assay was performed to evaluate the migration of HeLa cells after drug treatment. Briefly, HeLa cells were seeded in 6-well plates at 4 × 10^5^ cells per well and cultured till approximately 90% confluence. Afterward, the medium was removed and a 10 *µ*L pipette tip was used to generate a wound area by scraping the cell monolayer. The cells were treated with BCNU, BCNU + *O*^6^-BG or HACB/BCNU NPs under normoxia or hypoxia for 24 h. The concentration of BCNU in each group was 0.1 mM. For the BCNU + *O*^6^-BG group, the cells were pretreated with 40 *µ*M *O*^6^-BG for 2 h before exposure to BCNU. The wound width was observed and recorded by a fluorescent microscope (IX-51, Olympus Corporation, Tokyo, Japan). The cell migration rate was calculated according to Eq. (5) as follows:

5 $$\mathrm{Migration\, rate }(\mathrm{\%})=({S}_{0}-{S}_{24})/{S}_{0}\times 100$$

Here, *S*_24_ and *S*_0_ referred to the area of the region without cells at 24 and 0 h, respectively.

### Cell viability in spheroidal cultures

#### Tumor spheroid culture and morphology analysis

Three-dimensional (3D) tumor spheroids were established to mimic the in vivo hypoxic tumor microenvironment by culturing HeLa cells in a chitosan-hyaluronic acid (C-HA) scaffold, which was prepared according to the method described by Florczyk et al. [39]. HeLa cells were seeded onto the C-HA scaffold in 24-well plates at 5 × 10^4^ cells per scaffold and maintained for 1 h till the cell suspension penetrated the scaffold. Then, 1 mL complete medium was added to each well and the cells were incubated for 10 days at 37 ℃ and 5% CO_2_ with regular media changes every 2 days. The morphology of the HeLa spheroids inside the C-HA scaffold was acquired on a scanning electron microscope (SEM). The cell-cultured samples were fixed by 2.5% Karnovsky’s fixative overnight at 4 ℃ and dehydrated in a graded series of ethanol (0%, 30%, 50%, 75%, 95%, 100%). After that, the samples were critical-point dried and sputter-coated with gold before imaging with a SU3500 SEM (Hitachi, Tokyo, Japan) [10].

#### Inhibitory activity of HACB/BCNU NPs against tumor spheroids

HACB/BCNU NPs-induced inhibition of proliferation in tumor spheroids was assessed by Alamar blue assay. After 10 days of incubation, the samples were treated with BCNU, BCNU + *O*^6^-BG, HACB/BCNU NPs with proposed concentrations (50, 100, 200, 400 and 1000 *µ*M) in normoxia or hypoxia for 24 h. As described above, cells were exposed to 40 *µ*M *O*^6^-BG for 2 h before BCNU exposure for the combination-treated groups. The cells were washed with PBS followed by adding 500 *µ*L medium containing 10% Alamar blue. After incubation for 2 h, 100 *µ*L Alamar blue solution was transferred to a 96-well plate followed by measuring the absorbance at 570 nm and 630 nm. Ultimately, the cell viability (%) was calculated according to Eq. (6) as follows:

6 $$\mathrm{Cell\, viability }\left(\mathrm{\%}\right)=({E}_{630}\times {OD}_{T570}-{E}_{570}\times {OD}_{T630})/({E}_{630}\times {OD}_{NC570}-{E}_{570}\times {OD}_{NC630})\times 100$$

Here *E*_570_ and *E*_630_ refer to the absorption coefficient of Alamar blue at 570 nm and 630 nm, respectively. *OD*_T570_ and *OD*_T630_ are the absorption values of the drug-treated groups at 570 nm and 630 nm, respectively. *OD*_NC570_ and *OD*_NC630_ are the absorption values of the negative control groups at 570 nm and 630 nm, respectively [10].

### In vivo fluorescence imaging

In order to determine the bio-distribution of HACB/BCNU NPs, DiR-labelled HACB NPs were injected into the HeLa tumor xenograft mice via the tail vein followed by observation via a non-invasive near-infrared (NIR) optical imaging technique. When the tumor reached about 300 mm^3^, the HeLa tumor xenograft mice were randomly divided into three groups and treated with DiR and HACB/DiR NPs both at a dose of 1 mg DiR/kg via the tail vein. In addition, to further confirm the interaction between CD44 receptors and HA, the mice were administrated with a high dose of free HA (1200 mg/kg) via the tail vein 1 h before being treated with HACB/BCNU NPs. At the prearranged time points after injection, NIR fluorescent images were captured by an in vivo imaging system (IVIS, PerkinElmer, United States). Furthermore, major organs (including hearts, livers, spleens, lungs and kidneys) and tumors of each mouse were collected and imaged via the in vivo imaging system.

### In vivo synergistic therapeutic efficacy

Tumor growth inhibition of HACB/BCNU NPs was evaluated on HeLa tumor xenograft models. When the tumor reached about 100 mm^3^, the mice were randomly divided into five groups (five mice per group): (I) control group (PBS); (II) BCNU group at 15 mg BCNU/kg; (III) BCNU + *O*^6^-BG group (10 mg/kg of *O*^6^-BG was injected intraperitoneally 2 h before BCNU being injected through caudal vein at a dose of 15 mg/kg); (IV) HACB/BCNU NPs-low dose group at 5 mg BCNU/kg; (V) HACB/BCNU NPs-high dose group at 15 mg BCNU/kg. The body weights and the tumor volumes were recorded every 2 days. At day 15, the mice were sacrificed, and the tumors were collected and weighed. All tumors and major organs were fixed in 10% neutral buffered formalin for histological examination. TdT-mediated dUTP nick-end labeling (TUNEL) assay and hematoxylin and eosin (HE) assay were performed to further evaluate the antitumor effect of different formulations. Meanwhile, the major organs were also analyzed by the HE staining at the end of the treatment to determine the biocompatibility and adverse effect of formulations.

### Statistical analysis

The data are given as mean ± S. D.. The statistical significance was tested by a two-tailed Student’s *t*-test or one-way ANOVA. The values between groups were compared using Student’s *t*-test. A *p*-value of less than 0.05 was considered statistically significant.

## Results and discussion

### Synthesis and characterization of HACB conjugate

**Fig. 1:**
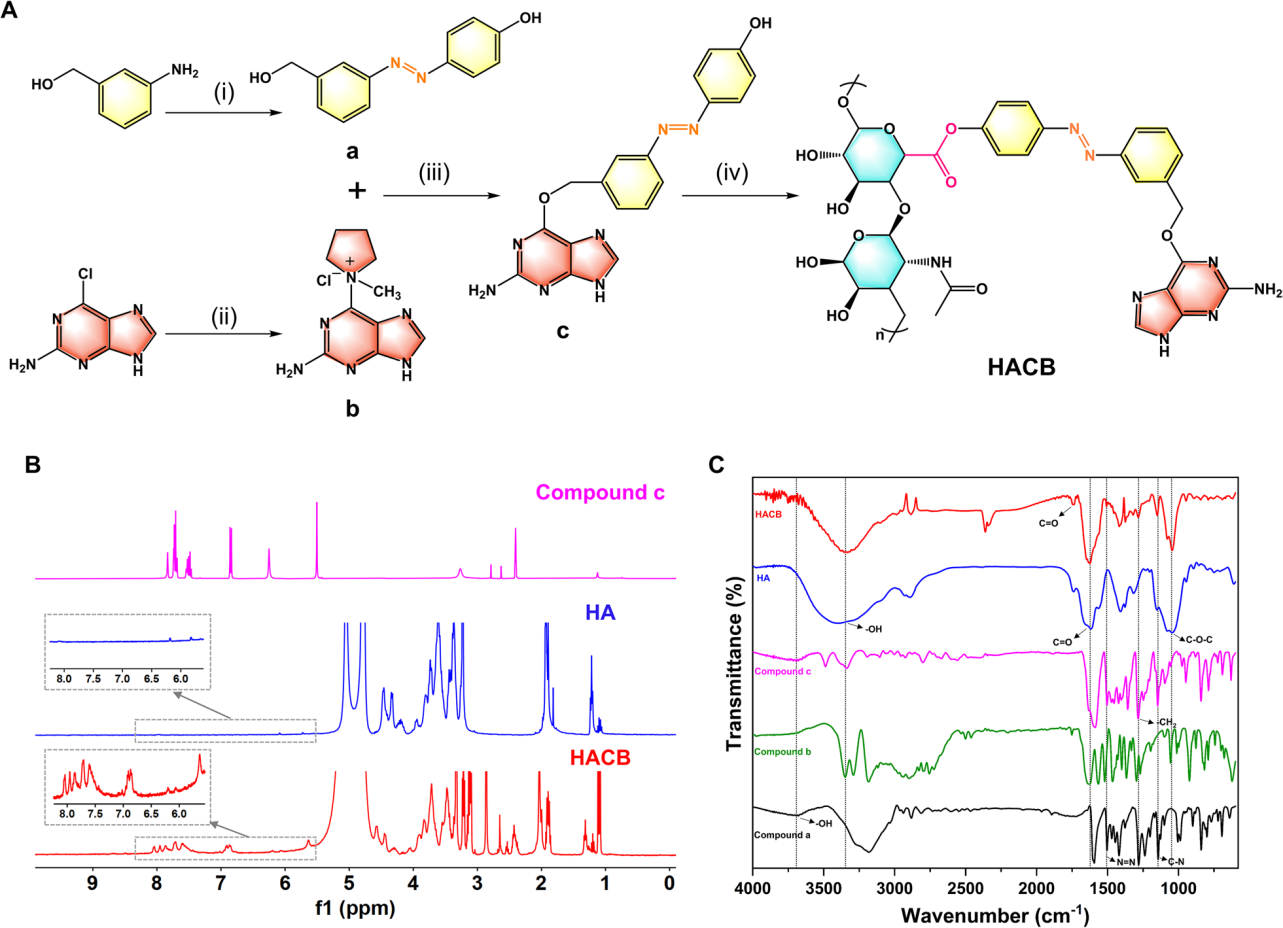
Synthesis, preparation and characterization of HACB. **A** Synthetic route of HACB. Reagents and conditions: (i) (1) H_2_O, HCl, NaNO_2_, 0–4 ℃; (2) Phenol, 0–4 ℃, 24 h. (ii) 1-Methylpyrrolidine, DMF, room temperature, 20 h. (iii) Tert-butoxide, DMF, nitrogen atmosphere, room temperature, 6 h. (iv) (1) HA, EDC, NHS, H_2_O, room temperature, 0.5 h; (2) Nitrogen atmosphere, 40 ℃, 48 h. **B** ^1^H NMR spectrum of compound c, HA and HACB: inset is a blown up of spectrum 5.5–8.2 ppm. **C** FT-IR spectrum of compound a, compound b, compound c, HA and HACB, from the bottom to the top

The synthesis procedure of HACB conjugate is depicted in Fig. 1A. Compound **a** was synthesized via diazotization between 3-aminobenzylalcohol and phenol. Then, compound **c** was formed by the condensation between compound **a** and compound **b** under the nitrogen atmosphere in presence of potassium tert-butoxide. Due to its azobenzene group, which undergoes azo bond cleavage and releases AGT inhibitor through electrons reduction under tumor hypoxic conditions, compound **c** is considered to be a hypoxia-degradable AGT inhibitor derivative [40–42]. Moreover, with abundant aromatic rings, the water-insoluble compound **c** acts as the hydrophobic terminal of HACB conjugate. Finally, amphiphilic conjugates HACB were synthesized by linking -COOH of HA and -OH of compound **c** with the esterification reaction. The chemical structure of the HACB conjugate was characterized and verified by ^1^H NMR and FT-IR. As shown in Fig. 1B, the peaks of HACB observed at δ = 1.8–2.1 ppm and δ = 6.5–8.2 ppm were assigned to -CH_3_ of HA and aromatic groups of compound **c**, respectively. Compared to the FT-IR spectrum of HA, the peak of HACB observed at 1730 cm^−1^ was assigned to stretching vibration of the carbonyl group from the newly formed ester (Fig. 1C). Moreover, the broad peak at 3330 cm^−1^ and the peak at 1043 cm^−1^ belonged to stretching vibration of –OH and C–O–C of HA, respectively. The peak at 1506 cm^−1^ was the characteristic peak of N=N group, and the peak at 1145 cm^−1^ was attributed to stretching vibration of C-N of azobenzene. These results together confirmed that HACB was successfully synthesized.

### Preparation and characterization of HACB NPs and HACB/BCNU NPs

**Fig. 2:**
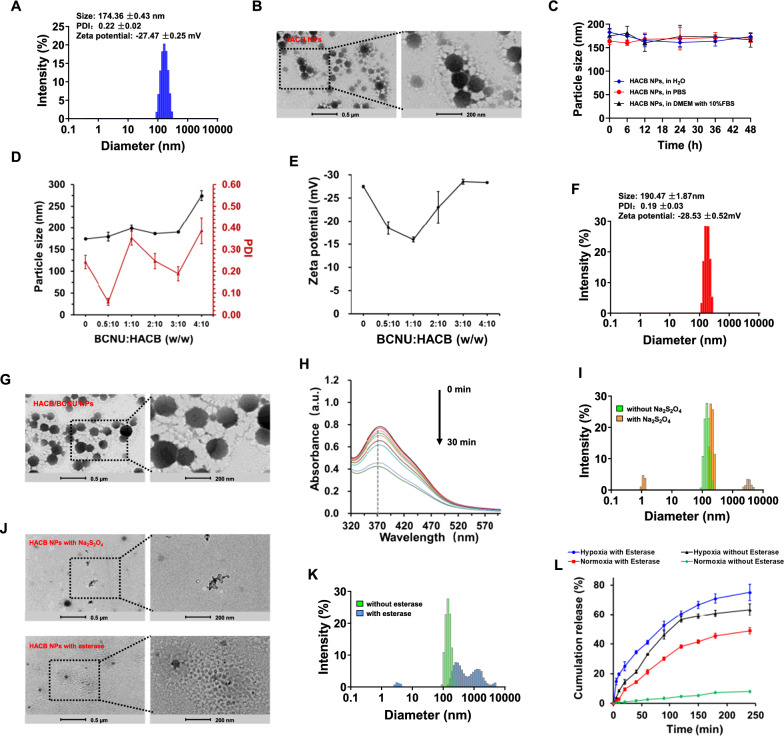
Characterization of HACB/BCNU NPs. **A** The hydrodynamic particle size of HACB NPs measured by dynamic light scattering. **B** TEM image of HACB NPs. Scale bars are 500 nm (left) and 200 nm (right). **C** The stability of HACB NPs. **D** The particle size and PDI of HACB/BCNU NPs. **E** Zeta potential of HACB/BCNU NPs. **F** The hydrodynamic particle size of HACB/BCNU NPs. **G** TEM image of HACB/BCNU NPs. Scale bars are 500 nm (left) and 200 nm (right). **H** The azo-benzene bond absorption changes of HACB NPs under reduction (Na_2_S_2_O_4_) at different time points. **I** The hydrodynamic particle size of HACB NPs after Na_2_S_2_O_4_ incubation. **J** TEM images of HACB NPs after Na_2_S_2_O_4_ or esterase incubation. Scale bars are 500 nm (left) and 200 nm (right). **K** The hydrodynamic particle size of HACB NPs after esterase incubation. **L** In vitro Cou6 release behavior of HACB/Cou6 NPs. Data are shown as mean ± S. D. (n = 3)

Due to its amphiphilic property, HACB conjugate could self-assemble into HACB micelles (HACB NPs) in an aqueous solution. As shown in Fig. 2A, the obtained HACB NPs presented a particle size of 174.36 ± 0.43 nm and polydispersity index (PDI) of 0.22 ± 0.02 with the zeta potential of − 27.47 ± 0.25 mV. From the TEM image shown in Fig. 2B, HACB NPs appeared a homogeneous spherical structure with an average diameter of 150–170 nm, which was consistent with the results of DLS. Moreover, HACB NPs retained its original size without any aggregation after incubation with H_2_O, PBS and DMEM containing 10%FBS, which confirmed the high stability of HACB NPs (Fig. 2C).

The particle size determines the tumor targeting ability and internalization efficiency of vehicles [43–45]. Hence, the particle size of HACB/BCNU NPs was chosen as the key indexes to optimize the synthesis conditions. Besides, the weight ratio of BCNU and HACB conjugate is another important factor to change the particle size of HACB/BCNU NPs. As shown in Fig. 2D, the particle size of HACB/BCNU NPs gradually fluctuated with the increasing weight ratio of BCNU and HACB conjugate. When the weight ratio of BCNU and HACB conjugate increased to 4: 10 (w/w), the particle size exceeded 250 nm. Additionally, the PDI values of HACB/BCNU NPs with a dosing ratio of 4: 10 were greater than 0.3, which were considered unstable in drug delivery applications. The optimal particle size (190.47 ± 1.87 nm) of HACB/BCNU NPs was achieved at 3:10 (w/w) with the PDI of 0.19 ± 0.03 (Fig. 2F). The zeta potential of HACB/BCNU NPs was − 28.53 ± 0.52 mV in this ratio (Fig. 2E). The high negative charge would increase the stability of vehicles in dispersion and make them more attracted to the surfaces of tumor cells [46, 47]. Moreover, the selection of micelles feeding weight ratio should ensure that BCNU-loaded micelles possess as high DL and EE as possible. As shown in Additional file 1: Table S1, when the weight ratio of BCNU and HACB conjugate increased to 3:10 (w/w), the EE and DL of HACB/BCNU NPs were up to 54.0 and 10.3%, respectively, which implied that the suitable feeding weight ratio was favorable for BCNU encapsulation. As observed by TEM, the morphology of HACB/BCNU NPs exhibited spherical structure without significant change after encapsulation of BCNU (Fig. 2G).

### The hypoxia/esterase-responsive of HACB NPs

#### Hypoxia-responsive dissociation of HACB NPs

To verify the responsiveness of HACB NPs to hypoxia, the hypoxic bioreductive environment was stimulated by the incubation of Na_2_S_2_O_4_ and HACB NPs in aqueous solution at 37 ℃ [48]. As shown in Fig. 2H, the characteristic absorption peak of azo at 370 nm presented a time-dependent disappearance, indicating that the azobenzene bridge in HACB NPs was successfully disrupted under hypoxic bioreductive environment. As shown in Fig. 2I and J, the hydrodynamic size of HACB NPs transformed into tri-modal distribution from original unimodal distribution and HACB NPs showed an irregular island-like morphology after Na_2_S_2_O_4_ treatment. The above data confirmed that HACB NPs possessed high hypoxia sensitivity and could be disintegrated via the breakage of azo bonds under hypoxia conditions, resulting in the changes of morphology and hydrodynamic size of the micelles.

#### Esterase responsiveness of HACB NPs

The phenyl hydroxyl ester structure in HACB NPs, which is a typical moiety undergoing rapid response to esterase [32], combines HA with the azobenzene-modified *O*^6^-BG and acts as an esterase-sensitive switch to control the drug release locating at the core of the micelles. To evaluate the esterase responsiveness of HACB NPs, we recorded the hydrodynamic size and morphology of the micelles after incubation with esterase by DLS and TEM, respectively. As shown in Fig. 2J and K, when subjected to esterase, the hydrodynamic size of HACB NPs was increased to more than 1000 nm and the intact spherical structure of the nanoparticles was destroyed. Therefore, these results suggested that esterase-induced uncoupling of the conjugates HACB eventually led to the disintegration of the micelles. The zeta potential of the nanoparticles after esterase treatment was measured, and the result showed that the treated micelles with the cleavage of ester bond still exhibited a negative potential without charge reversal (Additional file 1: Fig. S4).

### In vitro drug release profile

Cou6 is a classic model for the exploration of drug-releasing characteristics. Considering the poor stability of BCNU in aqueous solution, Cou6-loaded HACB NPs was prepared as an alternative of HACB/BCNU NPs. The release behavior of HACB/Cou6 NPs was analyzed by detecting the absorbance of Cou6 at 457 nm via HPLC. As shown in Fig. 2L, HACB/Cou6 NPs released only 8% over a period of 240 min under normoxia without esterase. While, in the presence of esterase, Cou6 was released from the HACB/Cou6 NPs rapidly with a cumulative release amount of 49% under normoxia, which was ascribed to the esterase-triggered rupture of the ester bonds in the micelles. Furthermore, hypoxic microenvironment facilitated the rapid release of Cou6 from HACB/Cou6 NPs. Over a period of 240 min under hypoxia, the cumulative release amount of Cou6 was 63% without esterase addition, while it reached to 75% with esterase addition, which was significantly higher (*p* < 0.05) than that of the group with esterase addition under normoxia. These results confirmed that the HACB/BCNU NPs were stable under normoxic physiological conditions, but the micelles could be disintegrated under high esterase content and hypoxic environment achieving rapid BCNU release in tumor tissues.

### Cellular uptake and intracellular tracking of HACB/Cou6 NPs

**Fig. 3:**
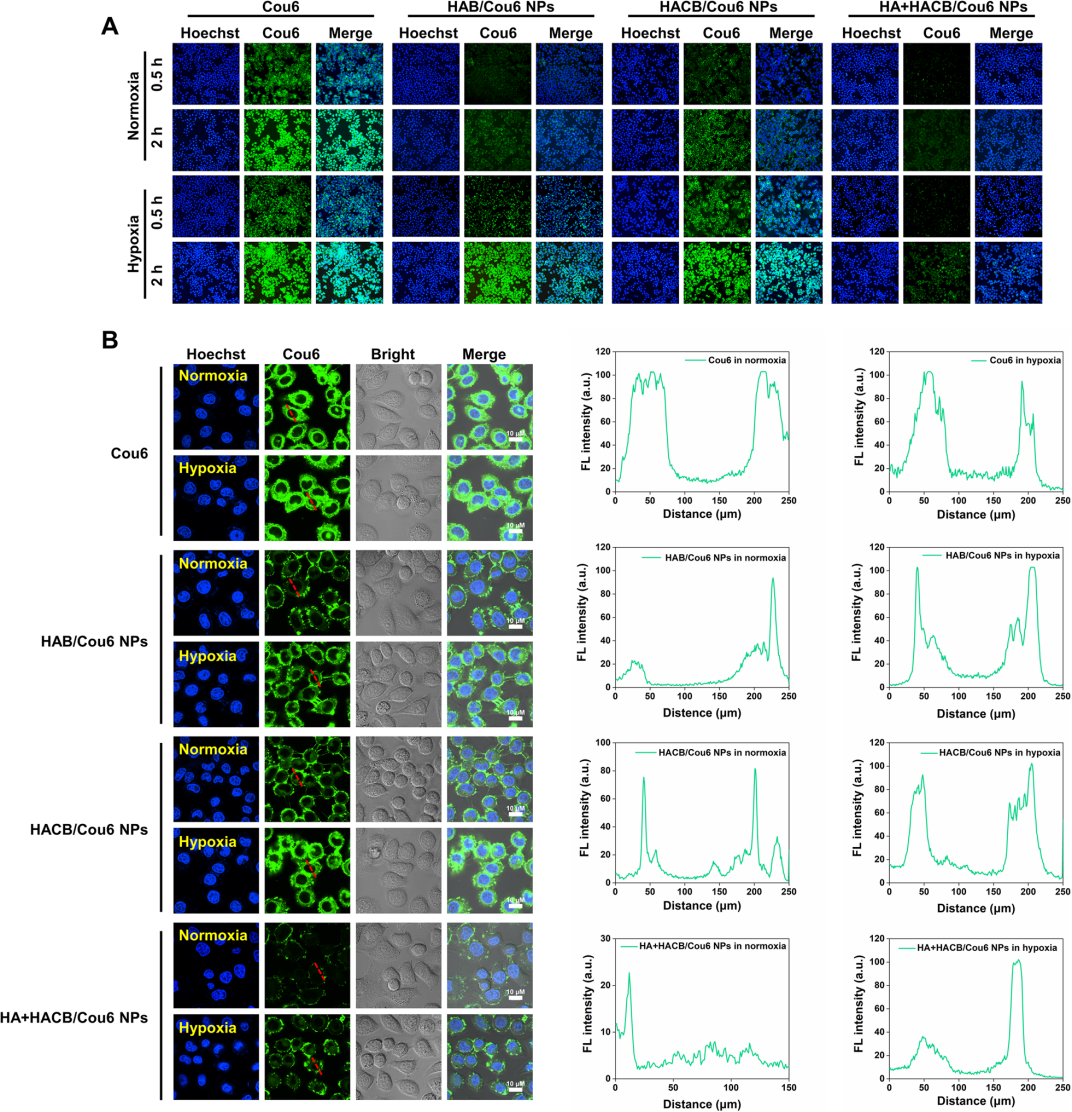
Uptake and intracellular localization of HACB/Cou6 NPs. **A** Cellular uptake of HeLa cells after treatment with Cou6, HAB/Cou6 NPs, HACB/Cou6 NPs and HA + HACB/Cou6 NPs under normoxia and hypoxia observed by fluorescence microscope (×100). **B** Fluorescence image of HeLa cells after 2 h treatment observed by confocal laser scanning microscopy. The profiles show the fluorescence of Cou6 in the area indicated by the red line. The scale bar is 10 *μ*m

The cellular uptake behavior of HACB/Cou6 NPs was observed by the fluorescent microscopic. Hoechst33342, a blue dye, was used to stain the nucleus for locating HeLa cells. Cou6 and HACB/Cou6 NPs were shown as green fluorescent. As illustrated in Fig. 3A, all groups showed enhanced green fluorescence signals with the extension of coincubation time. Similar green fluorescence signal intensity was observed for the normoxia and hypoxia groups treated by Cou6. While, stronger green fluorescence was observed in HeLa cells exposed to HA-AZO-BG/Cou6 (abbreviated as HAB/Cou6) NPs or HACB/Cou6 NPs under hypoxia than those under normoxia, indicating that azobenzene-based micelles could selectively dissociate and release Cou6 under hypoxia. It is noteworthy that the green fluorescence signal of HACB/Cou6 NPs group was stronger than HAB/Cou6 NPs group under normoxic conditions, which implied that HACB/Cou6 NPs entering HeLa cells could be hydrolyzed by the high content of esterase in the cells and release Cou6. Besides, the obvious weaker green fluorescence signal was observed in the HACB/Cou6 NPs groups with HA pretreatment than that in the HACB/Cou6 NPs groups without HA treatment, which could be due to the competitive blocking of HA-mediated active targeting, resulting in a significant reduction in cellular uptake of HACB/Cou6 NPs.

The intracellular localization of nano-micelles was further observed by CLSM (Fig. 3B). HACB/Cou6 NPs, which mainly distributed in the cytoplasm of HeLa cells, showed stronger green fluorescence compared with the HAB/Cou6 NPs under normoxia. In addition, the strongest green fluorescence signal was observed in HeLa cells exposed to HACB/Cou6 NPs under hypoxic conditions. Those results revealed that HACB/Cou6 NPs could be effectively internalized and accumulated in the cell cytoplasm, followed by degradation under the effect of hypoxia and high content of esterase in the cells.

### In vitro cytotoxicity assay

**Fig. 4:**
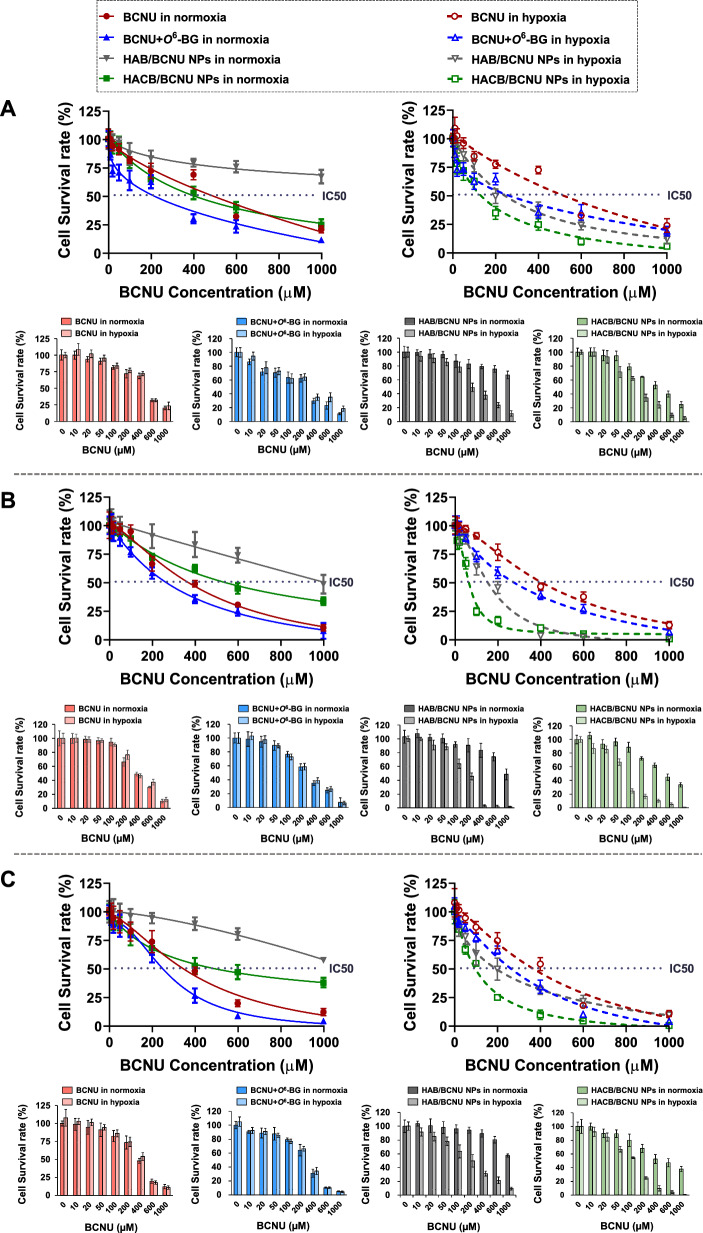
Survival rate of different cell lines after treatments. **A** HeLa, **B** A549, **C** SMMC-7721

In vitro anti-cancer efficacy of HACB/BCNU NPs was evaluated by MTT assay, colony-forming and live/dead staining assay. MTT assay was performed in HeLa, A549, and SMMC-7721 cells. The cell survival rate of all groups decreased with the increase of drug concentration (Fig. 4A–C). As depicted in Fig. 4A, HeLa cells treated with BCNU + *O*^6^-BG showed a lower survival rate compared with BCNU treatment. The IC_50_ values for the BCNU + *O*^6^-BG-treated groups were 213.2 *µ*M and 257.3 *µ*M in normoxia and hypoxia, respectively, which was lower than 489.5 *µ*M (normoxia) and 512.5 *µ*M (hypoxia) of the BCNU-treated groups (Additional file 1: Table S2). These results manifested that *O*^6^-BG could effectively sensitize tumor cells to BCNU by inhibiting the AGT activity.

**Fig. 5:**
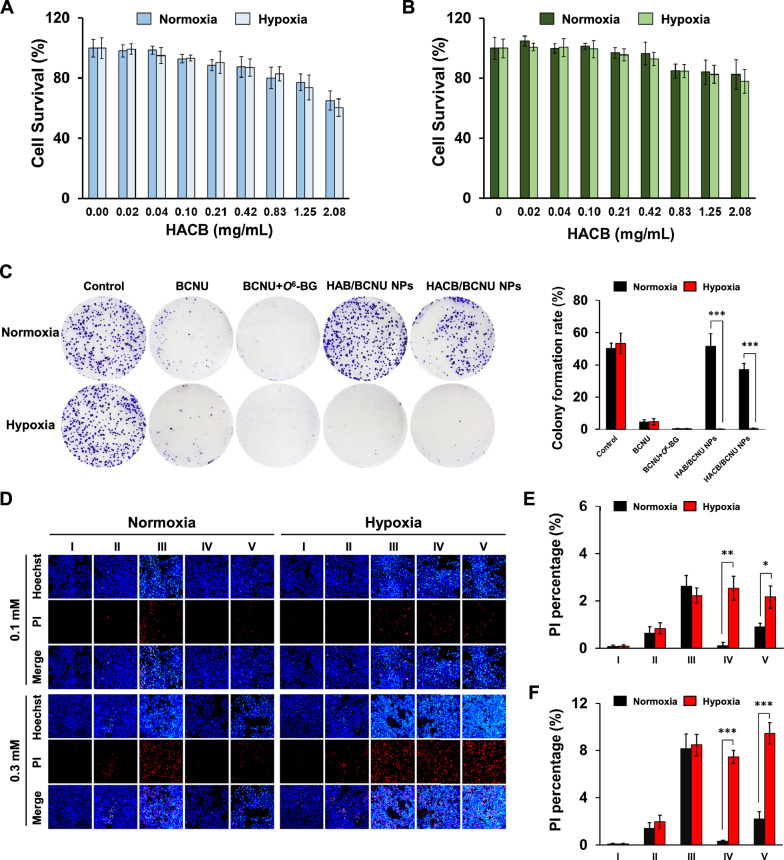
Cytotoxicity assay in vitro. **A**, **B** Survival rate of HeLa cells (**A**) and b End. 3 cells (**B**) treated with HACB NPs. **C** Colony formation images and colony formation rate were performed with HeLa cells after treatment. **D**–**F** The fluorescent images and dead population of HeLa cells after treatment observed by fluorescence microscope (×100). **E** Drug concentration of BCNU in each group is 0.1 mM; **F** Drug concentration of BCNU in each group is 0.3 mM. I Control, II BCNU, III BCNU + *O*^6^-BG, IV HAB/BCNU NPs, V HACB/BCNU NPs. Data are shown as mean ± S. D. (n = 3), *p < 0.05, **p < 0.01 and ***p < 0.001

Furthermore, no significant difference was observed in cell survival rate between the hypoxia and normoxia groups exposed to BCNU or BCNU + *O*^6^-BG (Fig. 4A). While, the cell survival rates of HeLa cells treated with azobenzene-based micelles (HAB/BCNU NPs groups and HACB/BCNU NPs groups) under hypoxia were significantly lower than those under normoxia. The IC_50_ values of HACB/BCNU NPs groups in normoxia and hypoxia were 401.4 *µ*M and 129.2 *µ*M, respectively, demonstrating the superior hypoxia selectivity of the micelles (Additional file 1: Table S2). In addition, compared to the HAB/BCNU NPs, HACB/BCNU NPs exhibited higher cytotoxicity against HeLa cells under normoxia. The IC_50_ value of HACB/BCNU NPs was 401.4 *µ*M under normoxia, which was significantly lower than HAB/BCNU NPs (> 1000 *µ*M) (Additional file 1: Table S2). This suggested that HAB/BCNU NPs with esterase-insensitive amide bonds was stable under normoxia and rarely caused drug leakage. While, BCNU could be released from the hydrolysis of the ester bonds in HACB/BCNU NPs by the high concentration of esterase in HeLa cells. Furthermore, it is worth noting that HACB/BCNU NPs exhibited about 3.8- and 1.9-fold stronger cytotoxicity than BCNU and BCNU + *O*^6^-BG under hypoxic conditions, respectively. This result could be attributed to the abundant *O*^6^-BG derivatives yielded by the fragmentation of the nano carriers via the cleavage of the azo bonds in HACB, thereby substantially sensitizing HeLa cells to BCNU. Similar results were obtained in A549 and SMMC-7721 cells (Fig. 4B and C). The biosafety of the blank carriers was investigated in HeLa cells and a normal cell line b End.3. As depicted in Fig. 5A and B, HACB NPs showed negligible cytotoxicity in HeLa and b End. 3 cells with more than 80% cellular viability at 0.83 mg/mL. Until the concentration of HACB NPs reached 2.08 mg/mL, the survival rates of HeLa and b End. 3 cells under hypoxic and normoxic conditions still exceeded 70%, showing only mild toxicity.

Figure 5C showed the colony formation inhibition of HeLa cells by the micelles. Under hypoxic conditions, HAB/BCNU NPs obviously inhibited the colony formation of Hela cells, while little inhibition effect was observed under normoxic conditions. However, the inhibition of HACB/BCNU NPs on HeLa colony formation was observed under both normoxic and hypoxic conditions. The above results are in accordance with the MTT assays, suggesting that HACB/BCNU NPs possessed strong inhibitory ability toward HeLa cell proliferation, especially in hypoxia. The live/dead staining assay was performed in HeLa cells to further assess the cellular injury induced by HACB/BCNU NPs. The apoptotic cells emitted a brighter fluorescence signal than the viable cells after stained with Hoechst 33342. The death cells showed red fluorescence after stained with PI. According to Fig. 5D, brighter blue and red fluorescence signal appeared in the group of HACB/BCNU NPs under normoxia compared with the group of HAB/BCNU NPs. Furthermore, HACB/BCNU NPs exhibited elevated activity inducing HeLa cells death under hypoxia than normoxia (Fig. 5E and F). Severe apoptosis and necrosis were observed in hypoxic HeLa cells exposed to high-dose HACB/BCNU NPs.

### Increase of apoptotic level by HACB/BCNU NPs

**Fig. 6:**
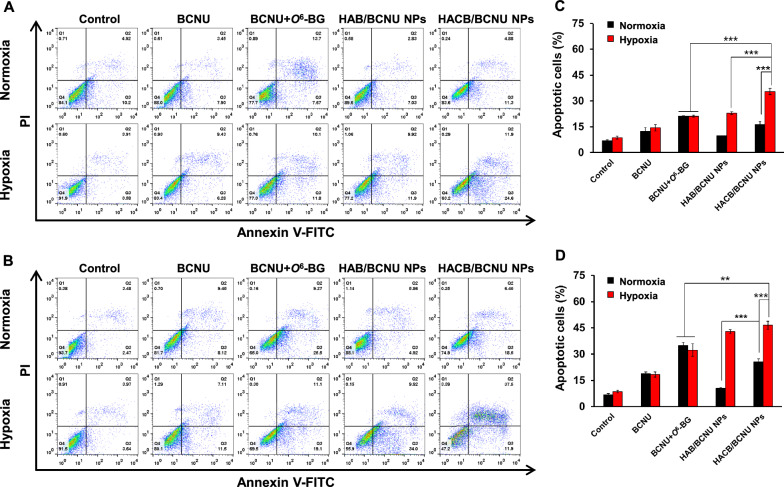
Flow cytometry analysis of HeLa cells after treatment. **A**, **C** Drug concentration of BCNU in each group is 0.1 mM; **B**, **D** Drug concentration of BCNU in each group is 0.3 mM. Data are shown as mean ± S. D. (n = 3), *p < 0.05, **p < 0.01 and ***p < 0.001

BCNU exerts antitumor activity by inducing the cancer cells to form the dG-dC ICLs, which causes cellular apoptosis by blocking the opening of DNA double strands in replication and transcription [49, 50]. Based on the results of cytotoxicity assays, we further analyzed the apoptosis level of HeLa cells induced by HACB/BCNU NPs. As shown in Fig. 6A and B, high-dose (0.3 mM) groups produced higher cellular apoptosis rates than their related low-dose (0.1 mM) groups. Under normoxic conditions, obvious enhanced apoptosis rate was observed in HeLa cells after HACB/BCNU NPs treatment compared with HAB/BCNU NPs. The cell apoptosis rate of HACB/BCNU NPs-group reach up to 16.30% at low-dose and 25.63% at high-dose, which was 1.67-fold (*p* < 0.001) and 2.4-fold (*p* < 0.001) higher than that of HAB/BCNU NPs-group, respectively (Fig. 6C and D). This result might be attributed to the successful hydrolysis of HACB/BCNU NPs by the esterase in HeLa cells. Moreover, the highest apoptosis ratio was observed in the HAB/BCNU NPs-treated cells under hypoxia. The cell apoptosis rates of HACB/BCNU NPs-treated groups under hypoxia rose to 35.48% at low-dose and 46.56% at high-dose, which were 1.68-fold (*p* < 0.001) and 1.44-fold (*p* < 0.01) higher than those of BCNU + *O*^6^-BG-treated group, respectively. These results confirmed that HACB/BCNU NPs could efficiently release BCNU and produce AGT inhibitors to induce apoptosis of HeLa cells under hypoxia.

### HACB/BCNU NPs inhibited the literal migration ability of HeLa cells

**Fig. 7:**
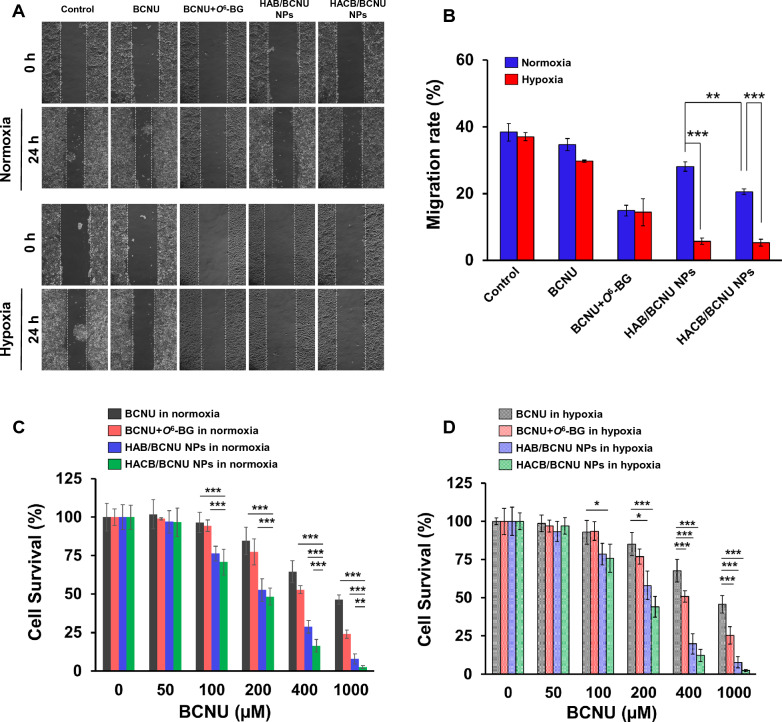
In vitro antitumor effect of HACB/BCNU NPs. **A**, **B** The migration of HeLa cells after treatment. **A**, **B** Indicates the visualization and quantified analysis of wound healing (×100). Data are shown as mean ± S. D. (n = 3). **C**, **D** Survival rate of HeLa cells in the tumor spheroids after treatment under normoxia (**C**) and hypoxia (**D**). Data are shown as mean ± S. D. (n = 6), *p < 0.05, **p < 0.01 and ***p < 0.001

To further evaluate the antitumor efficiency of HACB/BCNU NPs, the wound healing assay was carried out to investigate the inhibition of HACB/BCNU NPs on HeLa cell migration. As shown in Fig. 7A and B, no significant differences were observed between the cells treated with BCNU or BCNU + *O*^6^-BG under normoxic and hypoxic conditions. On the contrary, HACB/BCNU NPs exhibited superior hypoxia-responsive inhibition in HeLa cell migration. The cell migration ratio of HACB/BCNU NPs was 5.2% under hypoxia, obviously lower than that of BCNU (29.7%) and BCNU + *O*^6^-BG (14.4%).

### HACB/BCNU NPs inhibits the growth of tumor spheroids

As an in vitro model, 3D cell tumor spheroid is emerging as a promising platform for evaluating the anticancer activity of chemotherapeutic drugs. Three-dimensional (3D) tumor models possess similar characteristics to solid tumors, such as oxygen-deficient microenvironment, enhanced resistance and abnormal enzyme level [51]. In this study, 3D HeLa spheroids were cultivated on C-HA scaffolds to evaluate the antitumor ability of HACB/BCNU NPs. As shown in Fig. 7C and D, a dose-dependent decreased of cell survival rate was observed in all groups. HACB/BCNU NPs displayed notably enhanced inhibition against HeLa tumor spheroids compared with the other drug formulations. The IC_50_ of HACB/BCNU NPs in normoxia and hypoxia were 185.2 *µ*M and 175.2 *µ*M, respectively, which were remarkably lower (*p* < 0.001) than those treated with BCNU (800.8 *µ*M in normoxia and 805.8 *µ*M in hypoxia) and BCNU + *O*^6^-BG (442.4 *µ*M in normoxia and 392.4 *µ*M in hypoxia) (Additional file 1: Table S3).

Different from the results of 2D cells MTT assay, HACB/BCNU NPs presented superior inhibition against HeLa tumor spheroids under normoxia. This implied that the hypoxic and high enzyme content microenvironment formed by the 3D tumor model induced thorough fragmentation of the micelles, leading to complete drug leakage. In additions, due to the aggravation of drug resistance in HeLa tumor spheroids, the IC_50_ of BCNU and BCNU + *O*^6^-BG in HeLa tumor spheroids were higher than those of 2D adherent cells (Additional file 1: Table S3). While, the strong reversal effect on drug-resistance by HACB/BCNU NPs could still effectively sensitize and suppress HeLa cells, especially under hypoxic conditions.

### In vivo imaging analysis

**Fig. 8:**
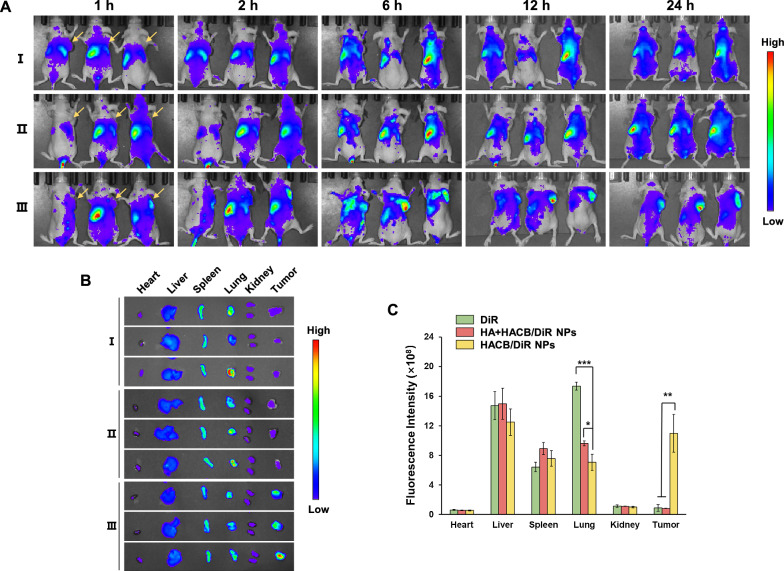
Tumor targetability of HACB NPs. **A** In vivo fluorescence imaging of HeLa tumor-bearing mice at 1, 2, 6, 12 and 24 h after intravenous injection of (I) free DiR, (II) HACB/DiR NPs with pre-injection of free HA and (III) HACB/DiR NPs. **B** Ex vivo fluorescence imaging of the tumor and normal tissues harvested from the euthanized HeLa tumor-bearing mice at 24 h post-injection. **C** Region-of-interest analysis of fluorescent signals from the tumors and normal tissues. Data are shown as mean ± S. D. (n = 3), *p < 0.05, **p < 0.01 and ***p < 0.001

To assess the tumor-targeting capability, the biological distribution of HACB/DiR NPs was visualized by non-invasive near-infrared fluorescence imagining technique. In vivo fluorescence images of free DiR and HACB/DiR NPs were monitored after injection within 24 h. As shown in Fig. 8A, HACB/DiR NPs displayed obvious fluorescence signal in the tumor region after 1 h injection, implying the rapid tumor targeting effect and efficient tumor-responsive release of HACB/DiR NPs. The obvious fluorescence signal was still observed after 24 h injection, demonstrating the persistent tumor retention effect of HACB/DiR NPs. On the contrary, free DiR was rapidly cleared after injection, and almost no fluorescence signal at the tumor site was captured within 24 h. To further verify the active-target ability of HACB/BCNU NPs, a high dose of HA was administrated intravenously before HACB/DiR NPs treatment to saturate CD44 receptors of tumor tissue. Then, a significant attenuation of fluorescence signal was observed in tumor tissue after HA + HACB/DiR NPs administration, illustrating that HACB/DiR NPs targeted tumors effectively.

After injection for 24 h, the mice were euthanized. The tumors and the major tissues were collected for ex vivo imaging. The fluorescence signal intensity was quantitative analyzed by region of interest (ROI). As illustrated in Fig. 8B, after 24 h injection, free DiR was mainly concentrated in the tissues of liver and lung, and negligible fluorescence signal was observed in the tumor tissues treated by free DiR. While for the HACB/DiR NPs group, the fluorescence signal was mainly concentrated in the tumor tissues, and the fluorescence intensity was 11.9- and 13.2-fold higher than those of the free DiR and HA + HACB/DiR NPs groups, respectively (Fig. 8C). These results suggested that the tumor targetability of the nanocarrier-based chemotherapy could effectively reduce the side effects of the traditional non-targeted BCNU.

### In vivo antitumor efficacy of HACB/BCNU NPs

**Fig. 9:**
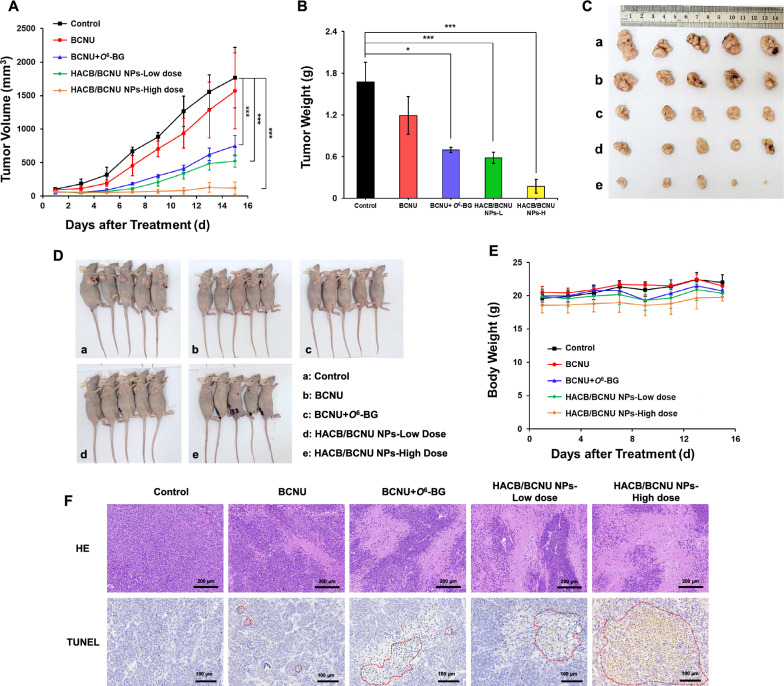
In vivo tumor therapeutic effect of HACB/BCNU NPs. **A** Tumor growth curves and **B** average tumor weight of the mice after injection with saline, BCNU, BCNU + *O*^6^-BG and HACB/BCNU NPs. **C** Image of tumors harvested after 15 days post-treatment. **D** Image of tumor-bearing mice after treatments. **E** The body weight variation of HeLa tumor-bearing mice during treatment. **F** Histological observation and detection of apoptosis in tumor tissues after treatment. The red circles indicate sites of apoptosis. Data are shown as mean ± S. D. (n = 5), *p < 0.05, **p < 0.01 and ***p < 0.001

To further evaluate the in vivo antitumor efficacy of HACB/BCNU NPs, HeLa tumor-bearing mice were treated with saline, free BCNU, BCNU + *O*^6^-BG, and HACB/BCNU NPs. As shown in Fig. 9A, free BCNU exhibited negligible suppression of tumor growth, while combination of BCNU with *O*^6^-BG showed an enhanced inhibitory effect (*p* < 0.05), but was still insufficient to completely eliminate HeLa tumors. It is noticeable that a higher inhibitory effect on tumor growth was achieved in the low-dose HACB/BCNU NPs group compared with BCNU + *O*^6^-BG group, which was attributed to the effective accumulation and degradability of the micelles on tumor tissue. Besides, the high-dose HACB/BCNU NPs group showed the strongest tumor inhibition potency. On day 15, the tumor weight of the high-dose HACB/BCNU NPs group was only 14.5% and 24.9% of the weight of BCNU and BCNU + *O*^6^-BG groups, respectively (Fig. 9B). As shown in Fig. 9C and D, the mice treated by high-dose HACB/BCNU NPs exhibited complete elimination of tumors at the end time point, indicating the superior therapeutic effect of HACB/BCNU NPs. Moreover, no significant weight loss was observed in all groups during treatment (Fig. 9E).

Subsequently, HE staining and TUNEL assay were performed to investigate the in vivo therapeutic efficacy of the five formulations. As showed in Fig. 9F, the typical pathological features of tumor cells including large, deep and irregularly shaped nuclei were observed in the negative control group. HACB/BCNU NPs treatment groups showed obvious tumor cell remission, such as tumor coagulative necrosis and nuclear fragmentation, especially in the high-dose group. Moreover, the high-dose HACB/BCNU NPs group displayed obvious morphological changes of apoptosis compared with the BCNU and BCNU + *O*^6^-BG groups.

**Fig. 10:**
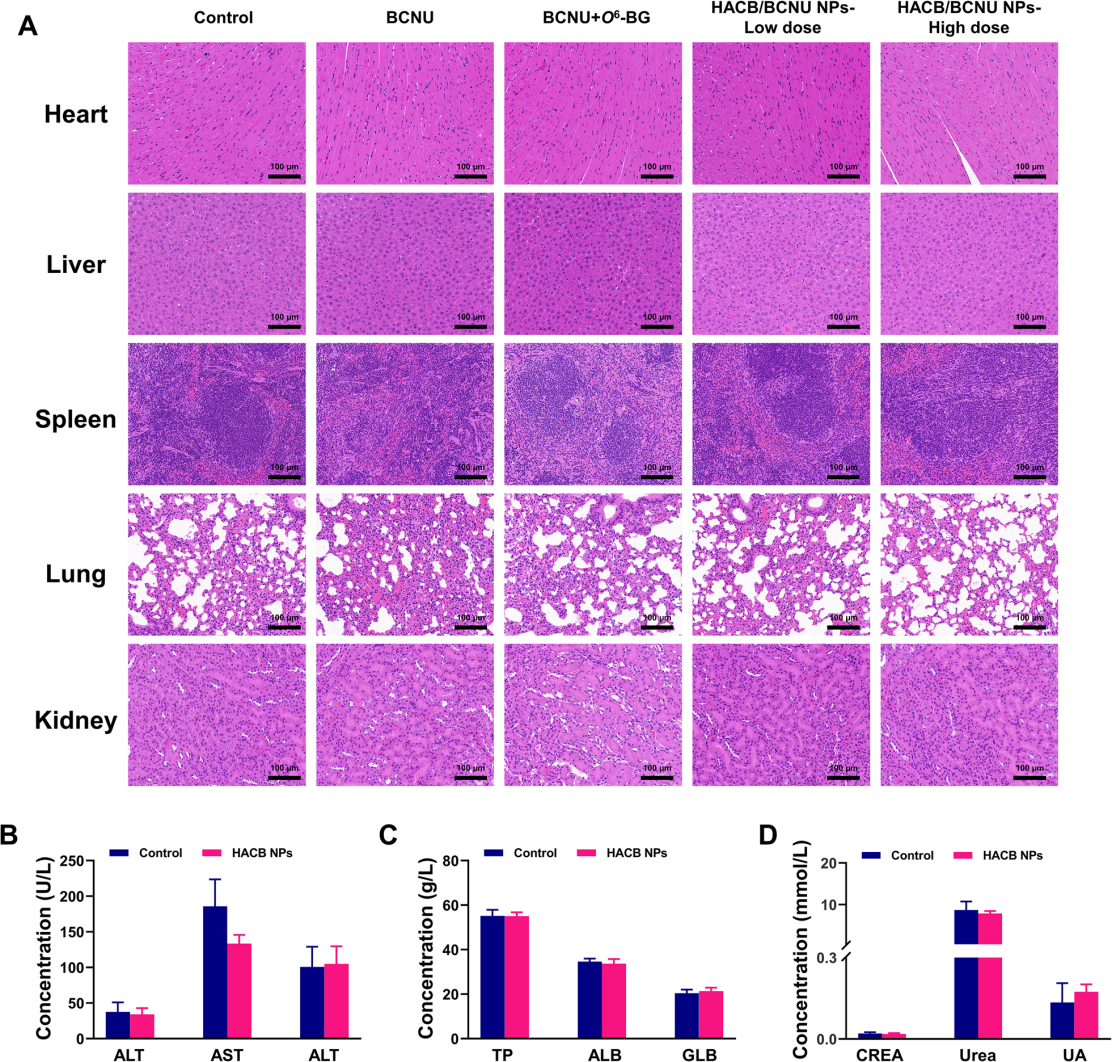
Biosafety evaluation in vivo. **A** Histological assessment of heart, liver, spleen, lung and kidney tissues of mice after treatment. The scale bar is 100 *µ*m. **B**–**D** Alanine aminotransferase (ALT), aspartate aminotransferase (AST), alkaline phosphatase (ALP), total protein (TP), albumin (ALB), globulin (GLB), creatinine (CREA), urea and uric acid (UA) levels from the mice treated with PBS and HACB NPs (1.5 mg/kg, i. v.) for 24 h. Data are shown as mean ± S. D. (n = 5)

The organs (heart, liver, spleen, lung and kidney) of the mice were excised for pathological examination to evaluate the biological toxicity of HACB/BCNU NPs. As showed in Fig. 10A, no obvious pathological change of toxicity was observed in all groups. In addition, the biosafety of HACB NPs was explored by performing the blood tests. As provided in Fig. 10B–D, all the biochemical blood indexes of HACB NPs group did not show abnormal changes compared to the control group, demonstrating that HACB NPs exhibited good biocompatibility and biosafety in vivo.

## Conclusion

In this study, a multifunctional hypoxia/esterase-sensitive nanomicelle was fabricated for the application of BCNU responsive delivery and tumor sensitization. The poor solubility and low stability of free BCNU in physiological buffers were improved by packaging BCNU in a spherical micelle with a hydrophilic surface. HACB/BCNU NPs could selectively disintegrate by azo bond cleavage under hypoxic conditions, leading to the emergence of AGT inhibitors and the release of BCNU. In vitro characterization experiment verified the good stability, high loading capacity of BCNU, HA-mediated high cell uptake and effective hypoxia/esterase responsiveness. By cytotoxicity assays, apoptosis analysis, wound-healing assays and in vivo antitumor assays, we observed that HACB/BCNU NPs exhibited significantly potentiated anticancer effect compared with conventional BCNU chemotherapeutics. Moreover, in vivo fluorescence imaging indicated that HACB NPs could specifically target tumor tissue. HACB/BCNU NPs showed favorable biocompatibility and biosafety in vivo. This high-performance stimulus-responsive nanocarrier paves a promising way for enhancing the anticancer efficacy and reducing the side effects of BCNU and other CENUs.

### Supplementary Information

**Additional file 1.** **Fig. S1** Chemical structural formula (S1.1) and ^1^H NMR (S1.2) and ^13^C NMR (S1.3) characterization of 4-((3-(hydroxymethyl) phenyl) diazenyl) phenol (a). **Fig. S2** Chemical structural formula (S2.1) and ^1^H NMR (S2.2) and ^13^C NMR (S2.3) characterization of 1-(2-amino-9*H*-purin-6-yl)-1-methylpyrrolidin-1-ium chloride (b). **Fig. S3** Chemical structural formula (S3.1) and ^1^H NMR (S3.2) and ^13^C NMR (S3.3) characterization of 4-((3-(((2-amino-9*H*-purin-6-yl) oxy) methyl) phenyl) diazenyl) phenol (c). **Fig. S4** The zeta pontential of HACB NPs after esterase incubation. **Table S1** Encapsulation efficiency and drug loading of HACB/BCNU NPs for different mass ratios of BCNU and HACB (w/w). **Table S2** The inhibitory concentration of each cell line after treatment. **Table S3** The inhibitory concentration of HeLa spheroids after treatment.

## Data Availability

Data will be made available on request.

## References

[1] Carter SK, Schabel FM, Broder LE, Johnston TP (1972). 1,3-bis(2-chloroethyl)-1-nitrosourea (bcnu) and other nitrosoureas in cancer treatment: a review. Adv Cancer Res.

[2] Yang MB, Tamargo RJ, Brem H (1989). Controlled delivery of 1,3-bis(2-chloroethyl)-1-nitrosourea from ethylene-vinyl acetate copolymer. Cancer Res.

[3] Burnham N, Betcher DL (1989). BCNU (carmustine). J Assoc Pediatr Oncol Nurses.

[4] Gnewuch CT, Sosnovsky G (1997). A critical appraisal of the evolution of N-nitrosoureas as anticancer drugs. Chem Rev.

[5] Kaina B, Christmann M (2019). DNA repair in personalized brain cancer therapy with temozolomide and nitrosoureas. DNA Repair.

[6] Nikolova T, Roos WP, Kramer OH, Strik HM, Kaina B (2017). Chloroethylating nitrosoureas in cancer therapy: DNA damage, repair and cell death signaling. Biochim Biophys Acta Rev Cancer.

[7] De Vita VT, Carbone PP, Owens AH, Gold GL, Krant MJ, Edmonson J (1965). Clinical trials with 1,3-bis(2-chloroethyl)-1-nitrosourea, NSC-409962. Cancer Res.

[8] Daniels DS, Woo TT, Luu KX, Noll DM, Clarke ND, Pegg AE, Tainer JA (2004). DNA binding and nucleotide flipping by the human DNA repair protein AGT. Nat Struct Mol Biol.

[9] Tubbs JL, Pegg AE, Tainer JA (2007). DNA binding, nucleotide flipping, and the helix-turn-helix motif in base repair by O^6^-alkylguanine-DNA alkyltransferase and its implications for cancer chemotherapy. DNA Repair (Amst).

[10] Liu Q, Wang X, Li J, Wang J, Sun G, Zhang N, Ren T, Zhao L, Zhong R (2021). Development and biological evaluation of AzoBGNU: A novel hypoxia-activated DNA crosslinking prodrug with AGT-inhibitory activity. Biomed Pharmacother.

[11] Pegg AE, Dolan ME, Moschel RC (1995). Structure, function, and inhibition of O^6^-alkylguanine-DNA alkyltransferase. Prog Nucleic Acid Res Mol Biol.

[12] Schilsky RL, Dolan ME, Bertucci D, Ewesuedo RB, Vogelzang NJ, Mani S, Wilson LR, Ratain MJ (2000). Phase I clinical and pharmacological study of O^6^-benzylguanine followed by carmustine in patients with advanced cancer. Clin Cancer Res.

[13] Friedman HS, Kokkinakis DM, Pluda J, Friedman AH, Cokgor I, Haglund MM, Ashley DM, Rich J, Dolan ME, Pegg AE (1998). Phase I trial of O^6^-benzylguanine for patients undergoing surgery for malignant glioma. J Clin Oncol.

[14] Quinn JA, Pluda J, Dolan ME, Delaney S, Kaplan R, Rich JN, Friedman AH, Reardon DA, Sampson JH, Colvin OM (2002). Phase II trial of carmustine plus O(6)-benzylguanine for patients with nitrosourea-resistant recurrent or progressive malignant glioma. J Clin Oncol.

[15] Wanner MJ, Koch M, Koomen GJ (2004). Synthesis and antitumor activity of methyltriazene prodrugs simultaneously releasing DNA-methylating agents and the antiresistance drug O(6)-benzylguanine. J Med Chem.

[16] Qiu Q, Domarkas J, Banerjee R, Merayo N, Brahimi F, McNamee JP, Gibbs BF, Jean-Claude BJ (2007). The combi-targeting concept: in vitro and in vivo fragmentation of a stable combi-nitrosourea engineered to interact with the epidermal growth factor receptor while remaining DNA reactive. Clin Cancer Res.

[17] Sun G, Zhang N, Zhao L, Fan T, Zhang S, Zhong R (2016). Synthesis and antitumor activity evaluation of a novel combi-nitrosourea prodrug: Designed to release a DNA cross-linking agent and an inhibitor of O(6)-alkylguanine-DNA alkyltransferase. Bioorg Med Chem.

[18] Wang Y, Ren T, Lai X, Sun G, Zhao L, Zhang N, Zhong R (2017). Synthesis and antitumor activity evaluation of a novel combi-nitrosourea prodrug: BGCNU. ACS Med Chem Lett.

[19] Jungk C, Chatziaslanidou D, Ahmadi R, Capper D, Bermejo JL, Exner J, von Deimling A, Herold-Mende C, Unterberg A (2016). Chemotherapy with BCNU in recurrent glioma: analysis of clinical outcome and side effects in chemotherapy-naive patients. BMC Cancer.

[20] Thambi T, Park JH, Lee DS (2016). Hypoxia-responsive nanocarriers for cancer imaging and therapy: recent approaches and future perspectives. Chem Commun.

[21] Yang S, Tang Z, Hu C, Zhang D, Shen N, Yu H, Chen X (2019). Selectively potentiating hypoxia levels by combretastatin A4 nanomedicine: toward highly enhanced hypoxia-activated prodrug tirapazamine therapy for metastatic tumors. Adv Mater.

[22] Zhang TX, Zhang ZZ, Yue YX, Hu XY, Huang F, Shi L, Liu Y, Guo DS (2020). A general hypoxia-responsive molecular container for tumor-targeted therapy. Adv Mater.

[23] Feng L, Cheng L, Dong Z, Tao D, Barnhart TE, Cai W, Chen M, Liu Z (2017). Theranostic liposomes with hypoxia-activated prodrug to effectively destruct hypoxic tumors post-photodynamic therapy. ACS Nano.

[24] Zhou H, Guo M, Li J, Qin F, Wang Y, Liu T, Liu J, Sabet ZF, Wang Y, Liu Y (2021). Hypoxia-triggered self-assembly of ultrasmall iron oxide nanoparticles to amplify the imaging signal of a tumor. J Am Chem Soc.

[25] Bae H, Jang JY, Choi SS, Lee JJ, Kim H, Jo A, Lee KJ, Choi JH, Suh SW, Park SB (2016). Mechanistic elucidation guided by covalent inhibitors for the development of anti-diabetic PPARgamma ligands. Chem Sci.

[26] Wang W, Lin L, Ma X, Wang B, Liu S, Yan X, Li S, Tian H, Yu X (2018). Light-induced hypoxia-triggered living nanocarriers for synergistic cancer therapy. ACS Appl Mater Interfaces.

[27] Zoller M (2011). CD44: can a cancer-initiating cell profit from an abundantly expressed molecule?. Nat Rev Cancer.

[28] Ossipov DA (2010). Nanostructured hyaluronic acid-based materials for active delivery to cancer. Expert Opin Drug Deliv.

[29] Li Y, Xiong J, Guo W, Jin Y, Miao W, Wang C, Zhang H, Hu Y, Huang H (2021). Decomposable black phosphorus nano-assembly for controlled delivery of cisplatin and inhibition of breast cancer metastasis. J Control Release.

[30] Sun Q, Bi H, Wang Z, Li C, Wang X, Xu J, Zhu H, Zhao R, He F, Gai S, Yang P (2019). Hyaluronic acid-targeted and pH-responsive drug delivery system based on metal-organic frameworks for efficient antitumor therapy. Biomaterials.

[31] Zhou Q, Mohammed F, Wang Y, Wang J, Lu N, Li J, Ge Z (2021). Hypoxia-responsive block copolymer polyprodrugs for complementary photodynamic-chemotherapy. J Control Release.

[32] Dong H, Pang L, Cong H, Shen Y, Yu B (2019). Application and design of esterase-responsive nanoparticles for cancer therapy. Drug Deliv.

[33] Lv L, Guo Y, Shen Y, Liu J, Zhang W, Zhou D, Guo S (2015). Intracellularly degradable, self-assembled amphiphilic block copolycurcumin nanoparticles for efficient in vivo cancer chemotherapy. Adv Healthc Mater.

[34] Qiu N, Liu X, Zhong Y, Zhou Z, Piao Y, Miao L, Zhang Q, Tang J, Huang L, Shen Y (2016). Esterase-activated charge-reversal polymer for fibroblast-exempt cancer gene therapy. Adv Mater.

[35] Huggins C, Moulton SH (1948). Esterases of testis and other tissues. J Exp Med.

[36] Su Y, Liu Y, Xu X, Zhou J, Xu L, Xu X, Wang D, Li M, Chen K, Wang W (2018). On-demand versatile prodrug nanomicelle for tumor-specific bioimaging and photothermal-chemo synergistic cancer therapy. ACS Appl Mater Interfaces.

[37] Keppler A, Gendreizig S, Gronemeyer T, Pick H, Vogel H, Johnsson K (2003). A general method for the covalent labeling of fusion proteins with small molecules in vivo. Nat Biotechnol.

[38] Zhang H, Li W, Guo X, Kong F, Wang Z, Zhu C, Luo L, Li Q, Yang J, Du Y, You J (2017). Specifically increased paclitaxel release in tumor and synergetic therapy by a hyaluronic acid-tocopherol nanomicelle. ACS Appl Mater Interfaces.

[39] Florczyk SJ, Wang K, Jana S, Wood DL, Sytsma SK, Sham J, Kievit FM, Zhang M (2013). Porous chitosan-hyaluronic acid scaffolds as a mimic of glioblastoma microenvironment ECM. Biomaterials.

[40] Nam S, Renganathan V (2000). Non-enzymatic reduction of azo dyes by NADH. Chemosphere.

[41] Zhou Y, Maiti M, Sharma A, Won M, Yu L, Miao LX, Shin J, Podder A, Bobba KN, Han J (2018). Azo-based small molecular hypoxia responsive theranostic for tumor-specific imaging and therapy. J Control Release.

[42] Zbaida S, Levine WG (1991). A novel application of cyclic voltammetry for direct investigation of metabolic intermediates in microsomal azo reduction. Chem Res Toxicol.

[43] Kaneda MM, Sasaki Y, Lanza GM, Milbrandt J, Wickline SA (2010). Mechanisms of nucleotide trafficking during siRNA delivery to endothelial cells using perfluorocarbon nanoemulsions. Biomaterials.

[44] Rejman J, Oberle V, Zuhorn IS, Hoekstra D (2004). Size-dependent internalization of particles via the pathways of clathrin- and caveolae-mediated endocytosis. Biochem J.

[45] Xiong H, Du S, Ni J, Zhou J, Yao J (2016). Mitochondria and nuclei dual-targeted heterogeneous hydroxyapatite nanoparticles for enhancing therapeutic efficacy of doxorubicin. Biomaterials.

[46] Wang Y, Guo M, Lu Y, Ding LY, Ron WT, Liu YQ, Song FF, Yu SQ (2012). Alpha-tocopheryl polyethylene glycol succinate-emulsified poly(lactic-co-glycolic acid) nanoparticles for reversal of multidrug resistance in vitro. Nanotechnology.

[47] Yin W, Qiang M, Ke W, Han Y, Mukerabigwi JF, Ge Z (2018). Hypoxia-responsive block copolymer radiosensitizers as anticancer drug nanocarriers for enhanced chemoradiotherapy of bulky solid tumors. Biomaterials.

[48] Long M, Lu A, Lu M, Weng L, Chen Q, Zhu L, Chen Z (2020). Azo-inserted responsive hybrid liposomes for hypoxia-specific drug delivery. Acta Biomater.

[49] Roos WP, Kaina B (2013). DNA damage-induced cell death: from specific DNA lesions to the DNA damage response and apoptosis. Cancer Lett.

[50] Nikolova T, Hennekes F, Bhatti A, Kaina B (2012). Chloroethylnitrosourea-induced cell death and genotoxicity: cell cycle dependence and the role of DNA double-strand breaks. HR and NHEJ Cell Cycle.

[51] Costa EC, Moreira AF, de Melo-Diogo D, Gaspar VM, Carvalho MP, Correia IJ (2016). 3D tumor spheroids: an overview on the tools and techniques used for their analysis. Biotechnol Adv.

