# Supplementary material for: A multi-functional hypoxia/esterase dual stimulus responsive and hyaluronic acid-based nanomicelle for targeting delivery of chloroethylnitrosouea
Source: J Nanobiotechnology. 2023 Aug 23;21:291. doi: 10.1186/s12951-023-02062-3 (PMC10464291; doi:10.1186/s12951-023-02062-3)
Supplement: Supplementary file 1 — Additional file 1. Fig. S1 Chemical structural formula (S1.1) and 1H NMR (S1.2) and 13C NMR (S1.3) characterization of 4-((3-(hydroxymethyl) phenyl) diazenyl) phenol (a). Fig. S2 Chemical structural formula (S2.1) and 1H NMR (S2.2) and 13C NMR (S2.3) characterization of 1-(2-amino-9H-purin-6-yl)-1-methylpyrrolidin-1-ium chloride (b). Fig. S3 Chemical structural formula (S3.1) and 1H NMR (S3.2) and 13C NMR (S3.3) characterization of 4-((3-(((2-amino-9H-purin-6-yl) oxy) methyl) phenyl) diazenyl) phenol (c). Fig. S4 The zeta pontential of HACB NPs after esterase incubation. Table S1 Encapsulation efficiency and drug loading of HACB/BCNU NPs for different mass ratios of BCNU and HACB (w/w). Table S2 The inhibitory concentration of each cell line after treatment. Table S3 The inhibitory concentration of HeLa spheroids after treatment. [file 12951_2023_2062_MOESM1_ESM.docx]

**Additional file**

**A multi-functional hypoxia/esterase dual stimulus responsive and hyaluronic acid-based nanomicelle for targeting delivery of chloroethylnitrosouea**

Duo Li, Ting Ren, Yunxuan Ge, Xiaoli Wang, Guohui Sun, Na Zhang, Lijiao Zhao *, Rugang Zhong

Beijing Key Laboratory of Environmental & Viral Oncology, Faculty of Environment & Life, Beijing University of Technology, Beijing 100124, China

**S1. Chemical structural formula and characterization of 4-((3-(hydroxymethyl) phenyl) diazenyl) phenol (a)**

S1.1 Chemical structural formula of compound **a**

S1.2 ^1^H NMR characterization of compound **a**

**
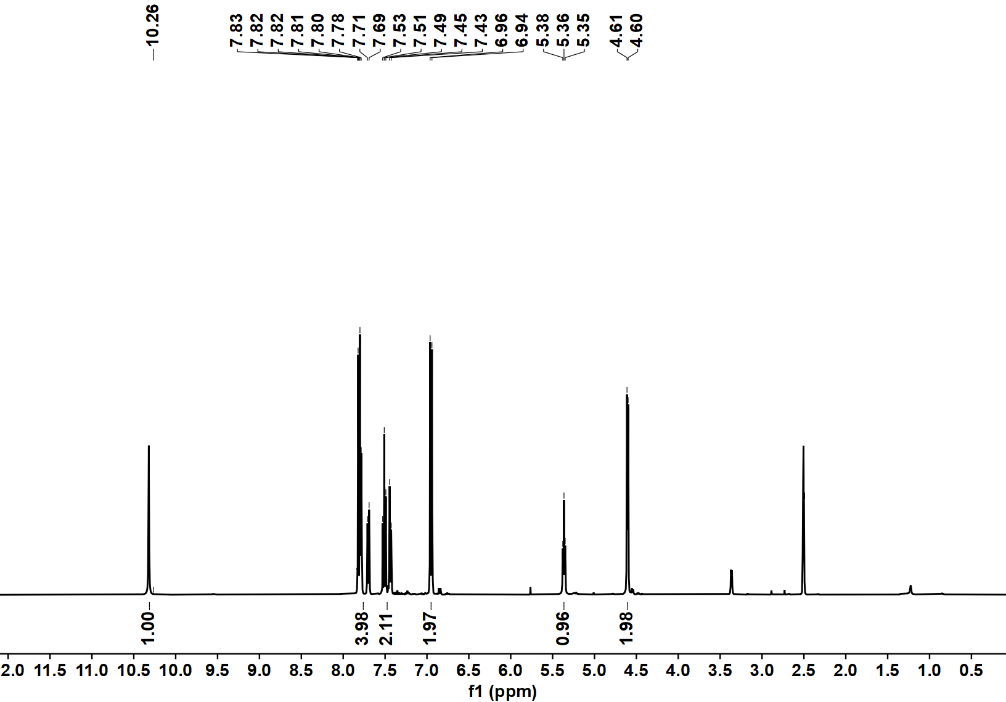
**

S1.3 ^13^C NMR characterization of compound **a**

**
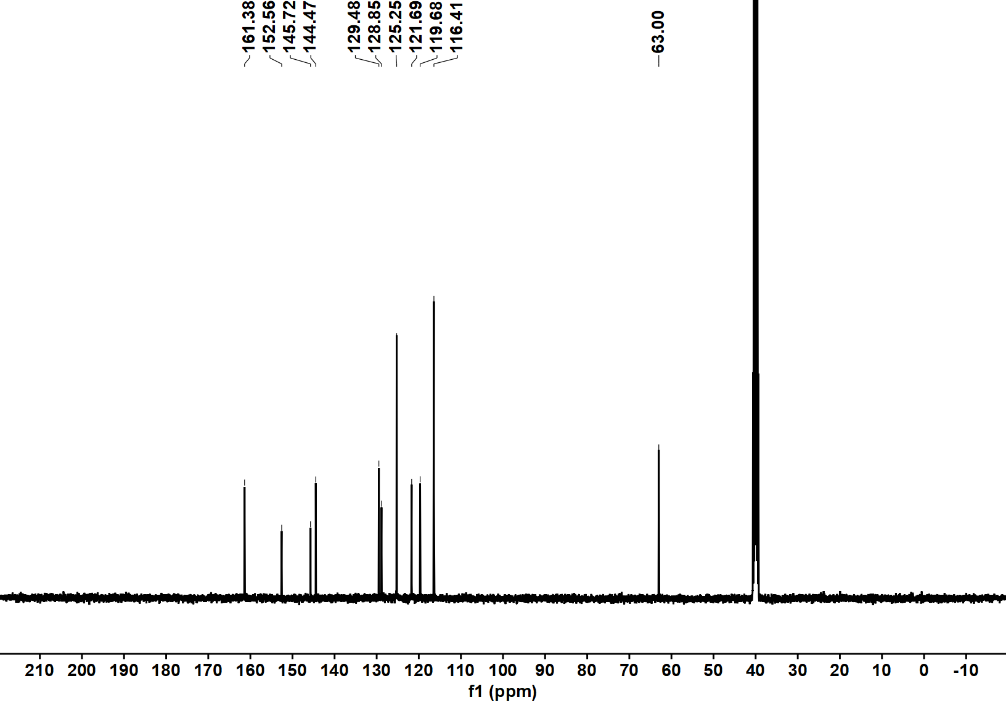
**

**S2. Chemical structural formula and characterization of 1-(2-amino-9H-purin-6-yl)-1-methylpyrrolidin-1-ium chloride (b)**

S2.1 Chemical structural formula of compound **b**

S2.2 ^1^H NMR characterization of compound **b**

**
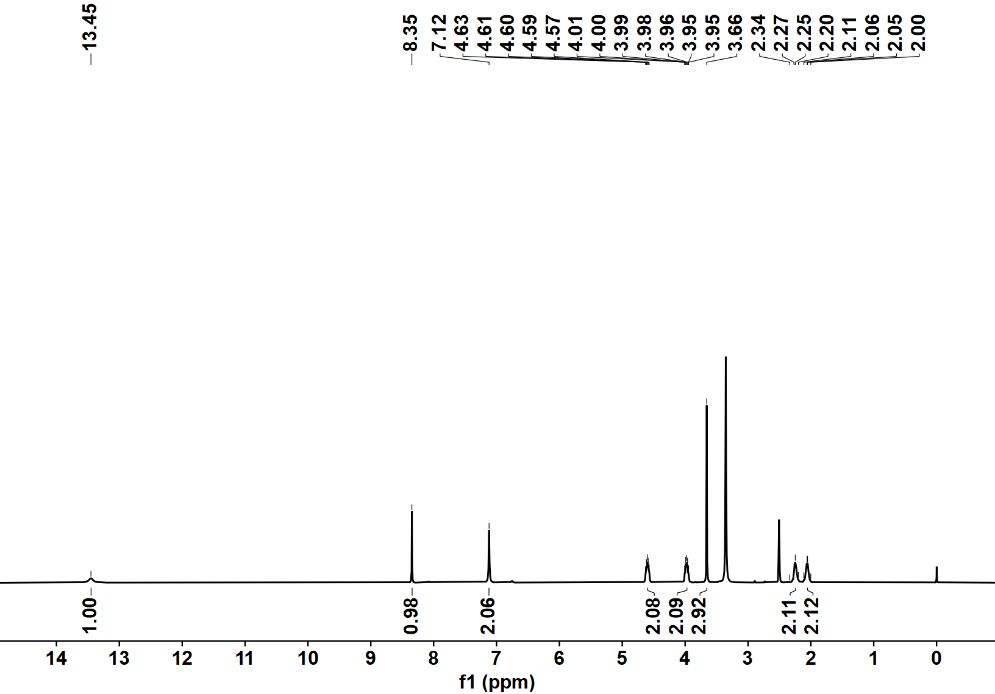
**

S2.3 ^13^C NMR characterization of compound **b**

**
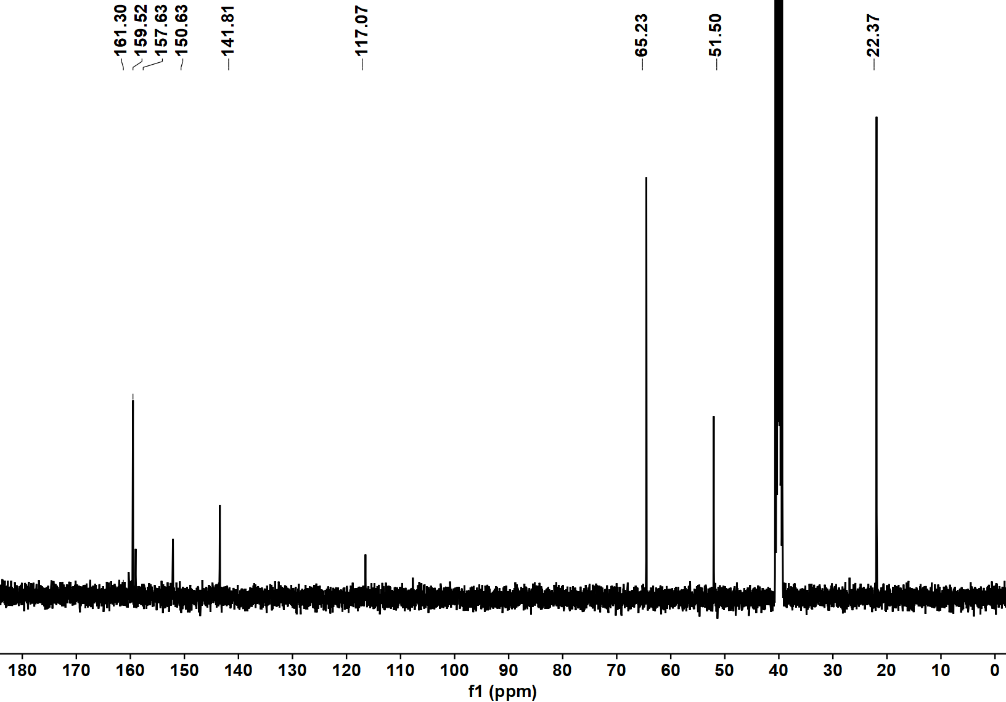
**

**S3. Chemical structural formula and characterization of 4-((3-(((2-amino-9*H*-purin-6-yl) oxy) methyl) phenyl) diazenyl) phenol (c)**

S3.1 Chemical structural formula of compound **c**

S3.2 ^1^H NMR characterization of compound **c**

**
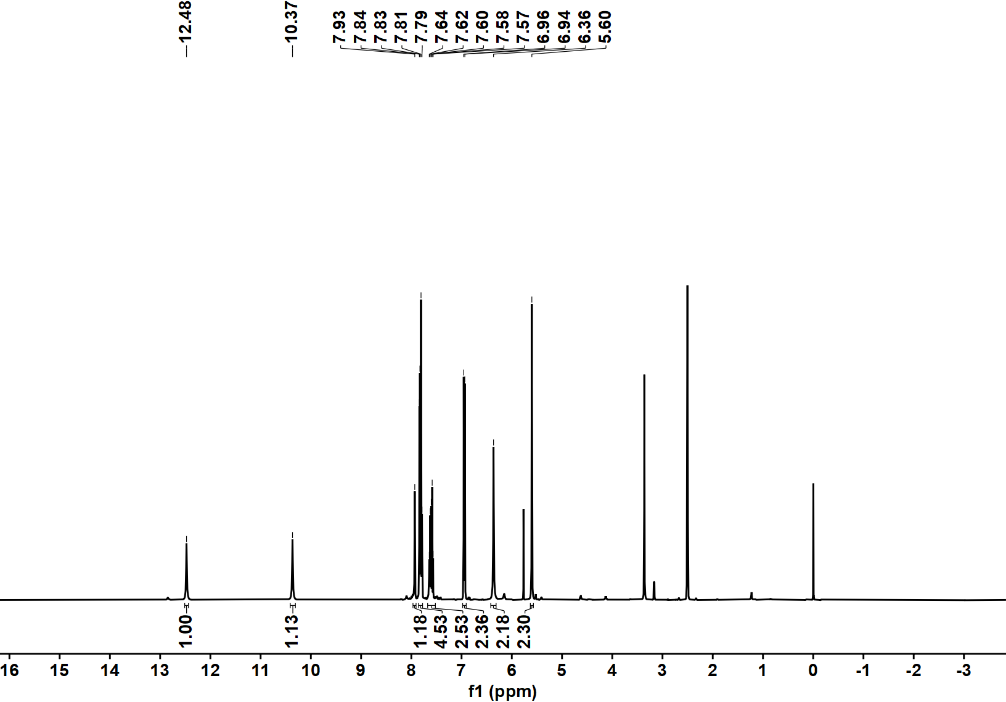
**

S3.3 ^13^C NMR characterization of compound **c**

**
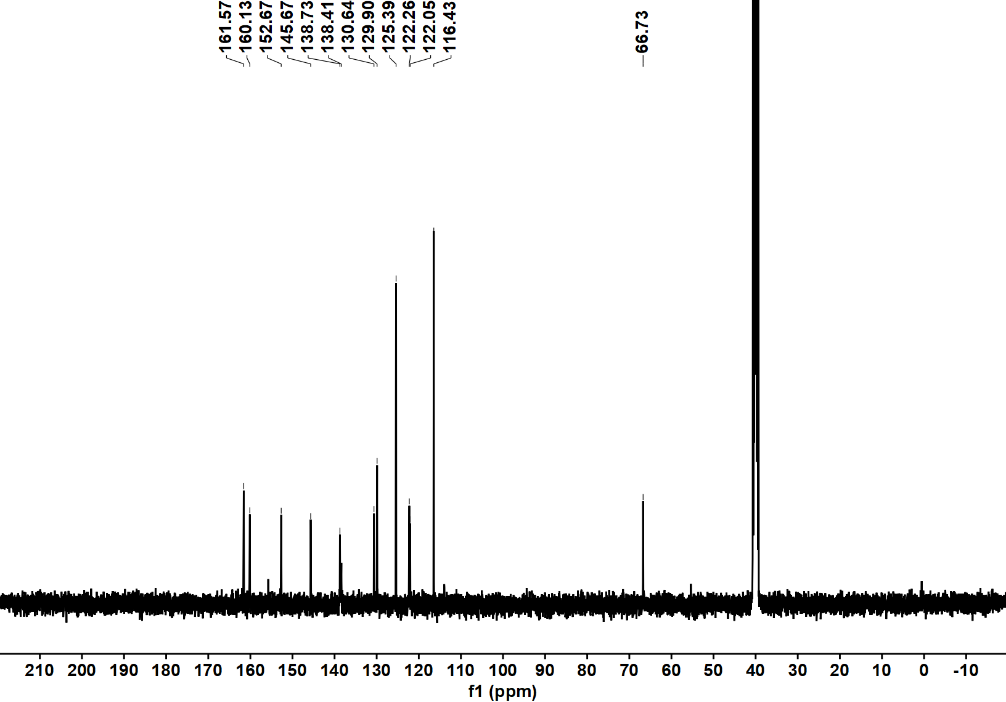
**

**S4 The zeta potential of HACB NPs after esterase incubation**

**Table S1.** Encapsulation efficiency and Drug loading of HACB/BCNU NPs for different mass ratios of BCNU and HACB (w/w)

| BCNU/HACB (w/w) | 0.5:10 | 1:10 | 2:10 | 3:10 | 4:10 |
| --- | --- | --- | --- | --- | --- |
| Encapsulation efficiency (EE, %) | 24.4 | 19.1 | 33.9 | 54.0 | 54.9 |
| Drug loading (DL, %) | 0.7 | 1.2 | 4.9 | 10.3 | 12.0 |

**Table S2.** The inhibitory concentration of each cell after treatment

| Cells | Drugs | IC_25_/µm | | IC_50_/µm | | IC_75_/µm | |
| --- | --- | --- | --- | --- | --- | --- | --- |
|  |  | Normoxia | Hypoxia | Normoxia | Hypoxia | Normoxia | Hypoxia |
| HeLa | BCNU | 186.2 | 229.2 | 489.5 | 512.5 | 880.9 | 917.9 |
|  | BCNU+*O^6^*-BG | 34.0 | 34.0 | 213.2 | 257.3 | 612.6 | 819.8 |
|  | HAB/BCNU NPs | 526.5 | 91.1 | ＞1000 | 240.2 | ＞1000 | 567.6 |
|  | HACB/BCNU NPs | 143.1 | 40.1 | 401.4 | 129.2 | ＞1000 | 349.3 |
| A549 | BCNU | 185.2 | 204.2 | 367.4 | 404.4 | 671.7 | 734.7 |
|  | BCNU+*O^6^*-BG | 108.1 | 106.1 | 264.3 | 274.3 | 561.6 | 590.6 |
|  | HAB/BCNU NPs | 516.5 | 82.1 | ＞1000 | 152.2 | ＞1000 | 266.3 |
|  | HACB/BCNU NPs | 204.2 | 35.0 | 538.5 | 64.1 | ＞1000 | 117.1 |
| SMMC-7721 | BCNU | 172.2 | 184.2 | 347.3 | 367.4 | 631.6 | 652.7 |
|  | BCNU+*O^6^*-BG | 138.1 | 119.2 | 250.3 | 264.3 | 416.4 | 494.5 |
|  | HAB/BCNU NPs | 691.7 | 54.1 | ＞1000 | 194.2 | ＞1000 | 524.5 |
|  | HACB/BCNU NPs | 133.1 | 38.0 | 497.5 | 97.1 | ＞1000 | 223.2 |

IC_25_= The inhibitory concentration 25%

IC_50_= The inhibitory concentration 50%

IC_75_= The inhibitory concentration 75%

**Table S3.** The inhibitory concentration of HeLa spheroids after treatment

|  | IC_25_/µM | | IC_50_/µM | | IC_75_/µM | |
| --- | --- | --- | --- | --- | --- | --- |
|  | Normaxia | Hypoxia | Normaxia | Hypoxia | Normaxia | Hypoxia |
| BCNU | 299.3 | 305.3 | 800.8 | 805.8 | ＞1000 | ＞1000 |
| BCNU+*O*^6^-BG | 238.2 | 195.2 | 442.4 | 392.4 | 978.9 | 997.9 |
| HAB/BCNU NPs | 112.1 | 123.1 | 216.2 | 220.2 | 427.4 | 381.4 |
| HACB/BCNU NPs | 106.1 | 107.1 | 185.2 | 175.2 | 320.3 | 285.3 |
